# Additive Blending Effects on PEDOT:PSS Composite Films for Wearable Organic Electrochemical Transistors

**DOI:** 10.1021/acsami.3c14961

**Published:** 2024-03-08

**Authors:** Hsueh-Sheng Tseng, Ying-Lin Chen, Pin-Yu Zhang, Yu-Sheng Hsiao

**Affiliations:** Department of Materials Science and Engineering, National Taiwan University of Science and Technology, Taipei 10607, Taiwan

**Keywords:** organic electrochemical transistors (OECTs), poly(3,4-ethylenedioxythiophene):poly(styrenesulfonate) (PEDOT:PSS), conductive polymers (CPs), nonionic fluorosurfactant (NIFS), self-healing

## Abstract

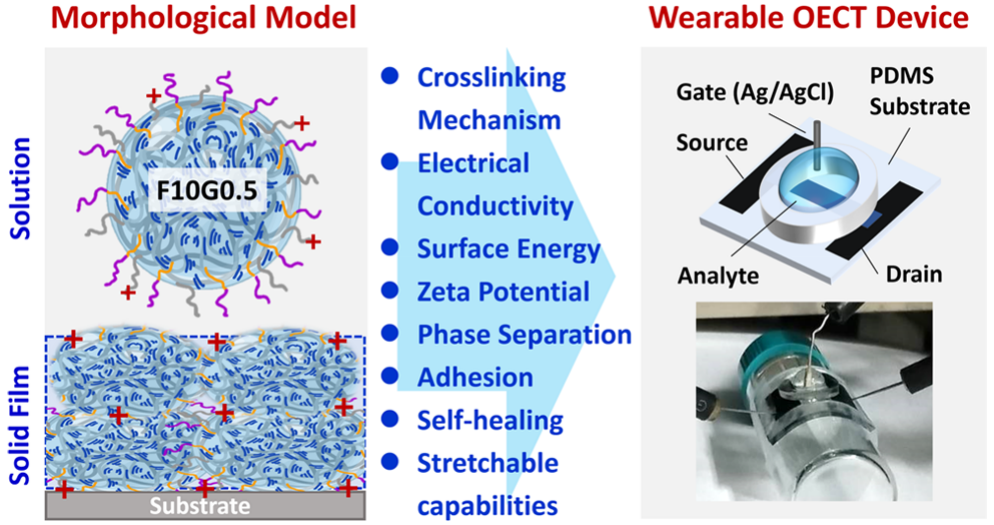

Organic electrochemical transistors (OECTs) employing conductive polymers (CPs) have gained remarkable prominence and have undergone extensive advancements in wearable and implantable bioelectronic applications in recent years. Among the diverse arrays of CPs, poly(3,4-ethylenedioxythiophene):poly(styrenesulfonate) (PEDOT:PSS) is a common choice for the active-layer channel in p-type OECTs, showing a remarkably high transconductance for the high amplification of signals in biosensing applications. This investigation focuses on the novel engineering of PEDOT:PSS composite materials by seamlessly integrating several additives, namely, dimethyl sulfoxide (DMSO), (3-glycidyloxypropyl)trimethoxysilane (GOPS), and a nonionic fluorosurfactant (NIFS), to fine-tune their electrical conductivity, self-healing capability, and stretchability. To elucidate the intricate influences of the DMSO, GOPS, and NIFS additives on the formation of PEDOT:PSS composite films, theoretical calculations were performed, encompassing the solubility parameters and surface energies of the constituent components of the NIFS, PEDOT, PSS, and PSS-GOPS polymers. Furthermore, we conducted a comprehensive array of material analyses, which reveal the intricacies of the phase separation phenomenon and its interaction with the materials’ characteristics. Our research identified the optimal composition for the PEDOT:PSS composite films, characterized by outstanding self-healing and stretchable capabilities. This composition has proven to be highly effective for constructing an active-layer channel in the form of OECT-based biosensors fabricated onto polydimethylsiloxane substrates for detecting dopamine. Overall, these findings represent significant progress in the application of PEDOT:PSS composite films in wearable bioelectronics and pave the way for the development of state-of-the-art biosensing technologies.

## 1 Introduction

Dopamine (DA), an essential neurotransmitter regulating neural communication within the central nervous system, plays a pivotal role in governing diverse human behavioral responses and cognitive functions.^1,2^ Disturbances in its equilibrium, whether excessive or deficient, have profound repercussions on human physiology, leading to motor impairments, cognitive decline,^3^ and the onset of neurological disorders, including attention deficit hyperactivity disorder (ADHD), Parkinson’s disease, Alzheimer’s disease, and schizophrenia.^4−7^ Given the formidable challenge and expense associated with detecting DA within the human brain coupled with its scant presence in body fluids (typically at levels of μM to nM),^8^ the development of wearable biosensors capable of attaining heightened sensitivity and rapid DA detection is of paramount importance for the accurate clinical diagnosis of associated neurological conditions. Quantifying DA concentrations in bodily fluids is a pivotal biomarker that enables real-time monitoring of neurological disorders by implementing convincing wearable biosensor technologies.

In recent years, wearable biosensors have garnered significant interest because of their ability to circumvent invasive detection and costly instrumentation. These sensors offer simple operation, affordability, ease of storage, and facile sample acquisition, greatly enhancing their utility in immediate and seamless personal health monitoring. Notably, wearable biosensors have witnessed significant advancements for diverse biomarkers found in human body fluids,^9−16^ including saliva,^17,18^ lactate,^19−21^ glucose,^22^ protein,^23^ uric acid (UA),^24,25^ sweat cortisol,^26^ and ions.^27,28^ Significant progress has also been made in the development of wearable sensors for DA detection and monitoring, capitalizing on their convenient storage, rapid detection capabilities, and enhanced sensitivity.^29^ Recent approaches employed for DA detection include spectroscopy, high-performance liquid chromatography, electrochemical techniques, and other analytical methods. Among these, electrochemical technologies have proven particularly advantageous for biosensing applications, attributed to their heightened sensitivity in detection, rapid response times, remarkable selectivity, and, in the case of DA, the ability to achieve a low limit of detection (LOD).^30−32^ Concurrently, the biosensing landscape has been enhanced by the growing adoption of organic electrochemical transistors (OECTs), which have garnered considerable interest for integration in biosensing applications. The prominent advantages of OECTs include cost-effective fabrication, enduring stability, heightened biocompatibility, and the ability to operate at remarkably low voltages (<1 V).^33^

Owing to its outstanding capacitive effect and electrochemical recognition, poly(3,4-ethylenedioxythiophene):poly(styrenesulfonate) (PEDOT:PSS) readily operates in the liquid state, offers a cost-effective solution (fabricated via a vacuum-free manufacturing process), demonstrates high biocompatibility, and maintains robust operational stability in aqueous environments. These attributes make the PEDOT:PSS film ideal as an active-layer channel within the OECT devices, effectively amplifying signals transduced between ions and electrons to meet the requirements of DA detection in biosensing applications. Moreover, the broad adoption of PEDOT:PSS-based OECT devices has been propelled by their remarkable transconductance and facile patterning, which makes them exceptionally versatile for various biosensor applications. A plethora of OECT-based biosensors have emerged, showcasing diverse functionalities, such as nanostructured OECT sensors for sweat cortisol detection,^26^ carbon grid electrode OECTs enabling ascorbic acid (AA) and DA detection,^34^ aerosol jet-printed OECTs for delta-9-tetrahydrocannabinol detection,^35^ paper-based flexible OECTs detecting glucose and H_2_O_2_,^36^ implantable OECTs for glucose and sucrose detection,^37^ OECT sensors for sialic acid detection,^38^ and nanobody-functionalized OECTs for protein detection.^39^

Recently, the demand for wearable biosensors has witnessed a notable upsurge. Given that wearable biosensors must withstand external abrasion, pulling, and mechanical bending, enhancing the viability of wearable OECTs requires the utilization of supple and robust active-layer materials to improve their stretchability.^40,41^ Notably, PEDOT:PSS films exhibit limited mechanical properties and are susceptible to damage. In this regard, the incorporation of additives like poly(dimethylsiloxane) (PDMS) elastomers or nonionic surfactants (such as nonionic fluorosurfactants [NIFS] or Triton X-100) into the PEDOT:PSS matrix has been explored to bolster their mechanical resilience.^42−46^ The addition of nonionic surfactants has proven effective in augmenting the stretchability and enhancing the viscoelastic properties of PEDOT:PSS hybrid films, albeit at the expense of a relative decrease in electrical conductivity. Although an alternative strategy involves introducing ionic liquids to foster a fibrous network within PEDOT:PSS to reorganize polymer chains and enhance stretchability,^47−49^ introducing these ionic liquids brings uncertainty to biosensing. Furthermore, the challenge of extending the lifespan of the active-layer materials in the development of OECTs remains a pressing concern, prompting investigations into imbuing biosensors with self-healing capabilities. Thus, a thorough analysis of PEDOT:PSS composite films will provide valuable insights into their morphological characteristics. This knowledge has the potential to significantly enhance the performance of wearable bioelectronics.

Therefore, this paper describes a comprehensive investigation of the impact of dimethyl sulfoxide (DMSO), (3-glycidyloxypropyl)trimethoxysilane (GOPS), and NIFS additives on the development of PEDOT:PSS composite films using the spin-coating process. Various physical properties associated with PEDOT:PSS-based composite solutions and films were analyzed including solubility parameters (δ) and surface energy (γ), determined through theoretical calculations of cohesive energy density (CED); viscosity measured using a rotational viscometer; electrical conductivity assessed via the four-point probe method; particle size distribution and ζ-potential determined through dynamic light scattering (DLS) and electrokinetic analysis (EKA); phase separation analyzed through atomic force microscopy (AFM), X-ray photoelectron spectroscopy (XPS), and depth profiles of time-of-flight secondary ion mass spectrometry (ToF-SIMS). Subsequently, a morphological model has been developed for PEDOT:PSS composite films, and their self-healing capabilities are evaluated. The optimized PEDOT:PSS composite films were also subjected to multiple cycles of tensile and rotational testing to demonstrate their mechanical durability. For example, the developed PEDOT:PSS composite films were applied to PDMS substrates and utilized as the active-layer channel of the OECTs. These films were specifically designed for seamless integration, with laser-scribed graphene (LSG) serving as the source and drain electrodes on the PDMS substrates. This integration aims to create a wearable OECT-based biosensor tailored to explore bioelectronic applications for DA detection.

## 2 Experimental Section

### 2.1 Materials

PEDOT:PSS aqueous solution (Clevios PH-1000) was purchased from Heraeus, Germany. DMSO, GOPS, DA, AA, and UA were purchased from Sigma-Aldrich. The PI tape was purchased from STAREK Scientific Co., Ltd. The PDMS prepolymers (SYLGARD 184A and 184B) were purchased from Sil-More Industrial, Ltd. The NIFS (Capstone FS3100) was obtained from DuPont Co. Ltd.

### 2.2 Preparation of PEDOT:PSS Composite Solutions

**Figure 1:**
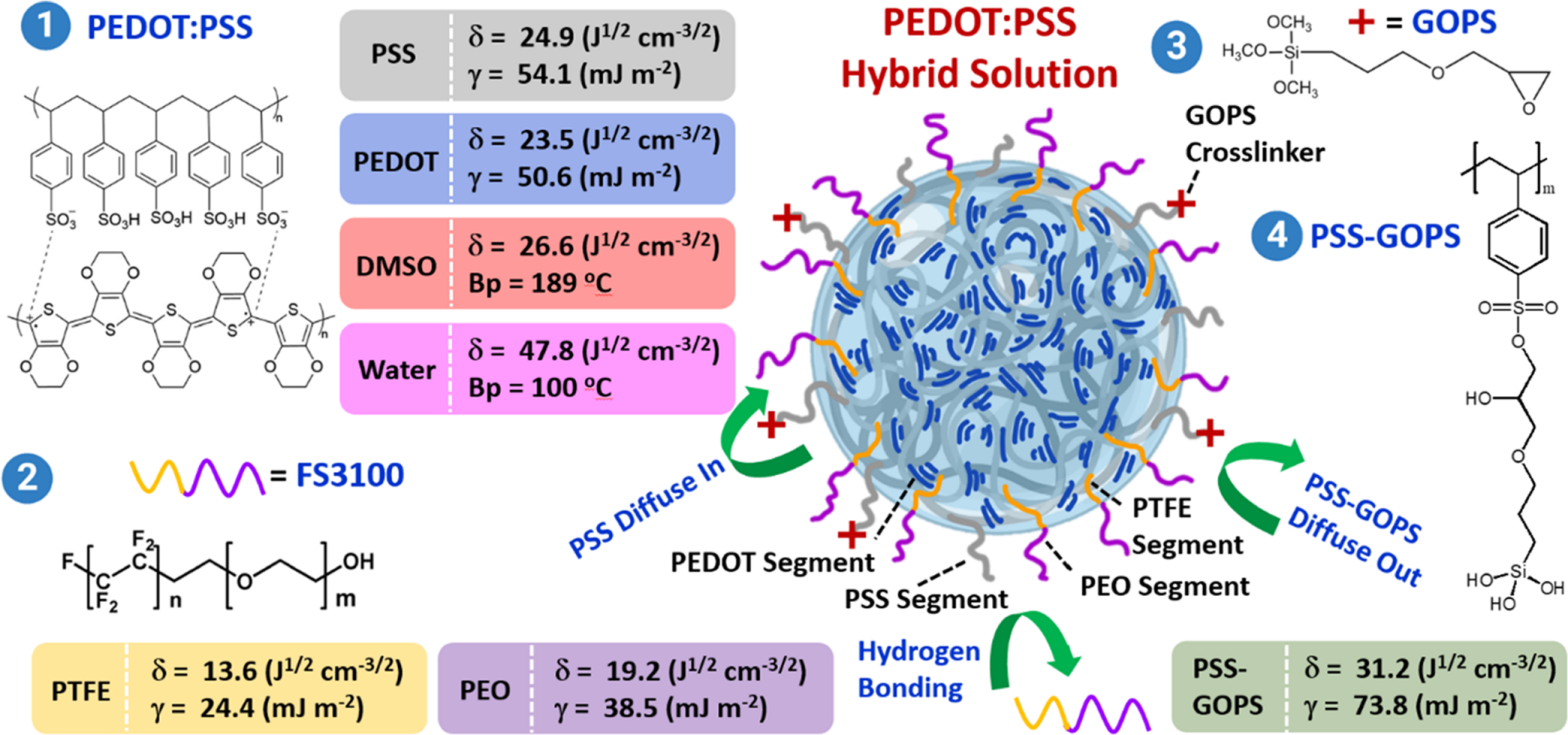
Schematic representation of PEDOT:PSS composite solutions with varying weight percentages of DMSO, GOPS, and FS3100 additives, along with the chemical structures of PEDOT, PSS, FS3100, GOPS, and PSS-GOPS, accompanied by their corresponding calculated solubility parameter (δ) and surface free energy (γ).

According to a previously reported procedure,^50^ aqueous PEDOT:PSS composite solutions were prepared by introducing varying FS3100 contents (0, 5, 10, 20, and 30 wt %), along with 5 wt % of DMSO and 1 wt % of GOPS, into the commercially available PEDOT:PSS dispersion (PH-1000) for thorough blending (Figure 1), denoted as **F0G1**, **F5G1**, **F10G1**, **F20G1**, and **F30G1**, respectively. An additional composition involving 10 wt % of FS3100, 5 wt % of DMSO, and 0.5 wt % of GOPS added to the PEDOT:PSS solution is referred to as **F10G0.5**. Before the spin-coating process, the composite solutions were stirred meticulously overnight by using a magnetic bar (at 800 rpm) and subsequently degassed by using a vacuum system for 15 min.

### 2.3 Characterization of the PEDOT:PSS Composite Materials

The dynamic viscosity of the PEDOT:PSS composite solutions was determined across a range of shear rates (3.7–93.0 s^–1^) through experimental analysis, utilizing a rotational viscometer (ViscoQC 300R, Anton Paar, Austria) set at 25 °C. The instrument was equipped with a cylindrical beaker (CC18). To assess the particle sizes and ζ-potentials of the PEDOT:PSS composite colloid solutions, DLS was performed using a Litesizer 500 instrument (Anton Paar, Austria).

To comprehensively characterize the PEDOT:PSS composite films, the following techniques were employed: (1) EKA measurements were performed by using a clamping cell connected to a SurPASS EKA analyzer (Anton Paar, Australia) with Au electrodes. This analysis facilitated the measurement of zeta-potentials (ζ-potentials) of the films at pH 7.4, utilizing a streaming current method and 0.01 M KCl as the electrolyte solution. (2) To determine the electrical conductivities of the films, a four-point probe and a Keithley 2400 source meter were used. (3) For surface morphology examination, AFM was employed to obtain topographic and phase images of the films using a Bruker Dimension Edge AFM operating in tapping mode at an ambient temperature. (4) XPS was conducted to analyze the chemical composition and phase separation of the films. The XPS spectra were recorded using a PHI 5000 VersaProbe system (ULVACPHI; Chigasaki, Japan) with a microfocused (100 μm, 25 W) Al Kα X-ray and a photoelectron takeoff angle of 45°. During spectral acquisition, a dual-beam charge neutralizer (7 V Ar^+^ and 1 V flooding electron beam) was employed to counteract the charge-up effect. (5) For comprehensive profiling and chemical analysis, a TOF-SIMS V spectrometer (ION-TOF, Germany) employing the time-of-flight secondary ion mass spectrometry (ToF-SIMS) techniques was utilized. This instrument was equipped with a 500 eV Cs^+^ sputter ion source and a 30 keV Bi_3_^+^ analysis source, enabling precise resolution of the chemical compositions within the PEDOT:PSS composite films. The process involved the initial use of a primary Cs^+^ ion beam (∼40 nA measured DC current) to raster 300 × 300 μm^2^ surface areas. Subsequently, a Bi_3_^+^ ion beam was employed to scan the crater centers, facilitating the collection of pertinent fragmented secondary ions. Depth profiles were obtained in noninterlaced mode, where sequential sputtering and analysis occurred under a base pressure of 10^–9^ Torr. In the negative spectra, the presence of secondary C^–^, 18O^–^, S^–^, Si^–^, and F^–^ ions (*m*/*z* 12, 18, 32, 28, and 19, respectively) was recorded as a characteristic of PEDOT:PSS composite films.

### 2.4 Self-Healing Capability Assessment of PEDOT:PSS Composite Films

Indium tin oxide (ITO) glass substrates (4.5 × 1.5 cm^2^) were cut and subsequently patterned using a CO_2_ laser system (Universal VLS2.30, Universal Laser System, AZ) with electrical tape (3M, 1350F-1) as a protection layer. This was followed by a wet-etching step using a standard 37% hydrochloric acid (HCl) solution to obtain the patterned ITO substrates. Next, a uniform 2 μm thick layer of the PEDOT:PSS composite film was deposited onto the patterned ITO glass substrates. The deposition was performed by using a spin-coating technique. The PEDOT:PSS composite solutions were used in the as-prepared state. To establish distinct PEDOT:PSS-based active-layer channels, a commercial CO_2_ laser engraving system was used on the deposited PEDOT:PSS composite films. The selection of a film thickness >1 μm was based on previous research,^51^ demonstrating improved and reliable self-healing capabilities under such conditions. The integrity of the PEDOT:PSS composite films was intentionally compromised by inducing damage with a blade knife. Subsequently, deionized (DI) water was applied to the impaired sections of the film. The self-healing ability of the PEDOT:PSS composite film was then assessed by monitoring the recovery of the electrical current. This was accomplished using a Keithley 2400 source meter unit that enabled precise current measurements.

### 2.5 Fabrication of OECT Devices on PDMS Substrates

First, PI tape was affixed to the surface of a 2 × 2 cm^2^ glass substrate. Next, two distinct LSG electrodes serving as the source and drain electrodes were patterned onto the PI tape. This patterning process was performed using a commercial CO_2_ laser engraving system (Universal VLS2.30, Universal Laser System, AZ), as previously described.^52^ Subsequently, the PDMS prepolymer was meticulously mixed to ensure a balanced 10:1 ratio of the base polymer to cross-linker. This well-mixed PDMS prepolymer solution (35 g) was then poured onto the LSG-patterned glass substrate situated within a 10 cm polystyrene dish, thus producing a 1 mm thick PDMS substrate incorporating the LSG electrodes. Following this, a degassing procedure was executed for 20 min and the mixture was cured in an oven at 60 °C for 6 h. Finally, the LSG electrodes were transferred from the PI tape to the PDMS substrate, resulting in the fabrication of patterned LSG/PDMS substrates measuring 2 × 2 cm each. The substrates were then stored in a drybox until further use.

To create an active-layer channel of PEDOT:PSS composite films approximately 200 nm in thickness on the LSG/PDMS substrate, a spin-coating process (4000 rpm, 20 s) was carried out with freshly prepared solutions. Subsequent to the spin-coating, thermal cross-linking was performed at 130 °C for 8 h. Then, the CO_2_ laser engraving system (Universal VLS2.30, Universal Laser System, AZ) was employed to eliminate undesired areas. Ultimately, the active-layer channel of the wearable OECT devices, with a width (W) of 1.5 mm and a length (L) of 5 mm, was encapsulated within a cylindrical PDMS chamber with a volume of approximately 65 μL. All electrical signals from the OECT devices were acquired in phosphate-buffered saline (PBS) (at a 1× concentration and pH 7.4). A silver/silver chloride (Ag/AgCl) wire was employed as the gate electrode and immersed in the test solutions to facilitate subsequent device characterization and biosensing applications.

## 3 Results and Discussion

### 3.1 Effect of Additives on PEDOT:PSS Composite Solutions

**Figure 2:**
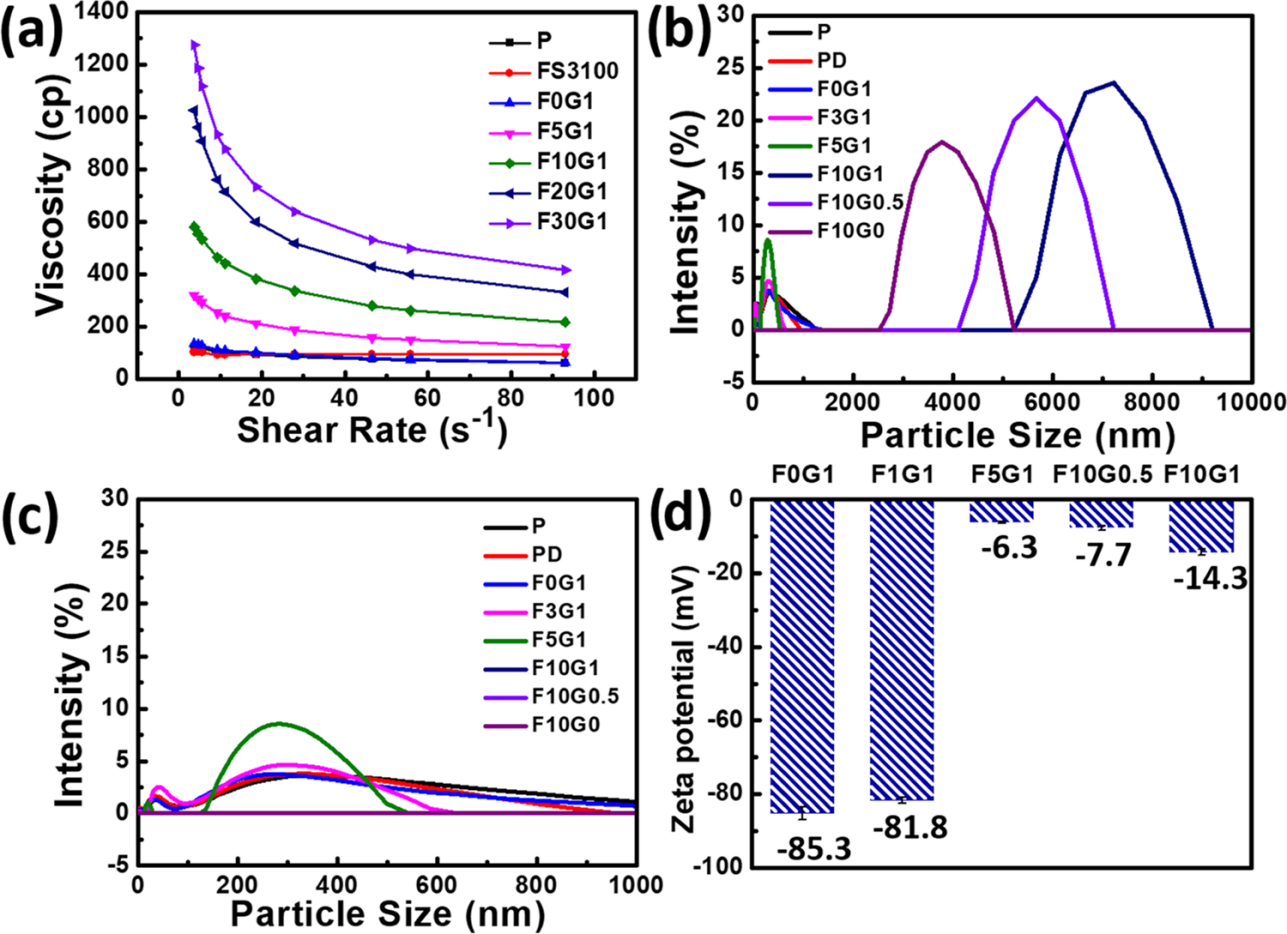
(a) Viscosities, (b, c) size distributions, and (d) ζ-potentials of PEDOT:PSS composite solutions with different DMSO, GOPS, and FS3100 additive contents, determined using a rotational viscometer, DLS, and ζ-potential measurements, respectively.

To investigate the effect of NIFS (such as FS3100) additives on the formation of PEDOT:PSS composite films in the presence of DMSO and GOPS using spin-coating, a comprehensive approach aimed at understanding the transition from a PEDOT:PSS solution to a solid-state film was adopted. Notably, the solubility parameter and surface energy calculated by PSS-GOPS (δ = 31.2 J^1/2^/cm^3/2^; γ = 73.8 mJ m^–2^, respectively) serve as valuable tools for elucidating and explaining the phase separation phenomenon observed in the transition of PEDOT:PSS composite materials from a solution to a solid film during the fabrication process.^53,54^ To gain insights into the domain-specific solubility and dispersion behaviors during the spin-coating process and to evaluate the hydrophilicity of PEDOT:PSS-based films, we conducted theoretical calculations concerning the solubility parameter and surface free energy for PSS, PEDOT, and PSS-GOPS polymers, as well as the PTFE and PEO segments within FS3100 (Figure 1). Utilizing estimated CED values across distinct structural groups, these calculations were informed by prior research, investigating nanophase separation within PEDOT-rich and PSS-rich domains.^53^ The solubility parameter and surface energy values derived from this calculation are as follows: PSS (δ = 24.9 J^1/2^/cm^3/2^; γ = 54.1 mJ m^–2^), PEDOT (δ = 23.5 J^1/2^/cm^3/2^; γ = 50.6 mJ m^–2^), PTFE (δ = 13.6 J^1/2^/cm^3/2^; γ = 24.4 mJ m^–2^), and PEO (δ = 19.2 J^1/2^/cm^3/2^; γ = 38.5 mJ m^–2^). For this, a commercial viscometer and DLS system were used to acquire data related to the viscosity, size distribution, and ζ-potential of the PEDOT:PSS colloid solutions with varying weight percentages of DMSO, GOPS, and FS3100 additives, as shown in Figure 2. Observations revealed that each PEDOT:PSS composite solution incorporating the FS3100 additive displayed pseudoplastic behavior within a specific shear rate range typically between 3 and 100 s^–1^ (Figure 2a). The incorporation of FS3100 resulted in a distinct and notable increase in viscosity, with values shifting from approximately 100 cP (cp) for **P**, **FS3100**, and **F0G1** to higher viscosities of approximately 180, 300, 400, and 500 cP for **F5G1**, **F10G1**, **F20G1**, and **F30G1**, respectively, all of which were measured at a shear rate of approximately 100 s^–1^. Given that FS3100 is an amphiphilic NIFS capable of interacting with PEDOT and PSS through the electronegative fluorine bonding and hydrogen bonding, respectively,^55^ the notable increase in viscosity suggests a more pronounced formation of OH···F hydrogen-bonded networks when higher quantities of FS3100 components are introduced into the PSS and PSS-GOPS domains of the **F0G1** solution.

The consistent results obtained from the DLS measurements substantiate the notion that introducing an extra 1 wt % GOPS during the transition from **F10G0** to both **F10G0.5** and **F10G1** results in an increase in the particle size of the PEDOT:PSS colloids, as shown in Figure 2b and Table S1. Similarly, the addition of extra FS3100 during the shift from **F0G1** to **F3G1**, **F5G1**, and **F10G1** also led to a noticeable increase in the particle size, as illustrated in Figure 2c and Table S1. Furthermore, ζ-potential measurements were conducted on a range of PEDOT:PSS composite solutions by using the same DLS system. This enabled the effect of the presence of GOPS and FS3100 on the surface charge characteristics of the PEDOT:PSS colloids to be assessed, as illustrated in Figure 2d. As previously documented,^54,56^ the inclusion of GOPS in an acidic PEDOT:PSS solution initiates the opening of epoxy rings, resulting in the generation of hydroxyl groups that interact with PSS. This interaction fosters the formation of non-cross-linked PSS-GOPS domains, involving the hydrolysis of the organylalkoxy group within the inorganic segments of the silane side chains. Notably, the introduction of 1 wt % GOPS into the PEDOT:PSS solution proved highly effective in generating a high surface energy of PSS-GOPS (γ = 73.8 mJ m^–2^). This establishment of robust OH···F (between PSS-GOPS and PTFE) and/or OH···O (between PSS-GOPS and PEO) hydrogen-bonded networks—due to a combination with the low surface energy of FS3100 (PTFE, γ = 24.1 mJ m^–2^; PEO, γ = 38.5 mJ m^–2^)—resulted in a reduced presence of PSS on the colloid surface. Consequently, this led to a significant reduction in the ζ-potential, shifting from −85.3 mV for **F0G1** to −6.3 mV for **F5G1**. Moreover, with the FS3100 content increasing from 5 to 10 wt %, the negative charge intensified further, eventually reaching −14.3 mV for **F10G1**. This raises the question of whether further increasing the FS3100 content could result in phase separation driven by the enhanced amphiphilic properties of FS3100 and its interactions with PEDOT and PSS.^57^ This interaction may cause the negatively charged PSS to migrate to the outer surface of the PEDOT:PSS colloids.

### 3.2 Effect of Additives on PEDOT:PSS Composite Films

**Figure 3:**
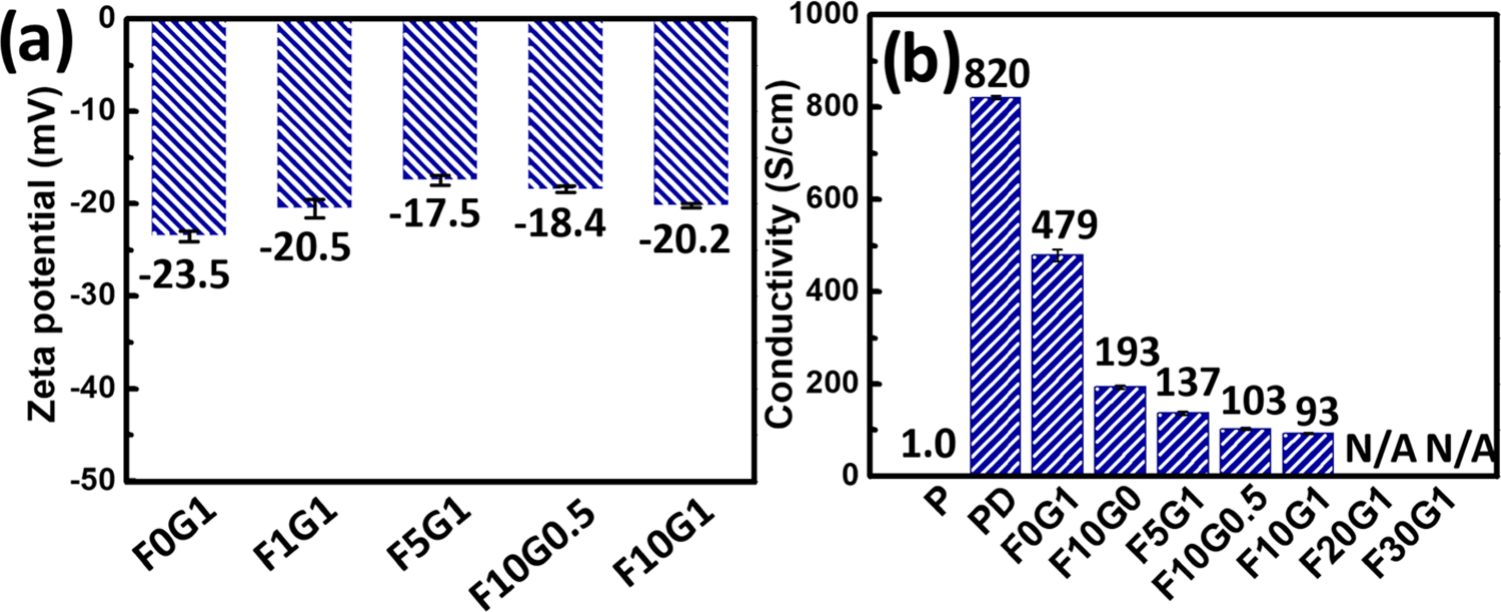
(a) ζ-Potentials and (b) electrical conductivity of PEDOT:PSS composite films with different DMSO, GOPS, and FS3100 additive contents, determined using electrokinetic and four-point probe measurements, respectively.

Figure 3a highlights the convergence of ζ-potentials of PEDOT:PSS composite films within a relatively narrow range (ranging from −17.5 to −23.5 mV). This range is in stark contrast to that of PEDOT:PSS-based colloid solutions, which exhibit a higher negative charge when applied to solid PEDOT:PSS composite films (Figure 2d). Thus, it is evident that there is significant phase separation, with the negatively charged PSS migrating to the outer surface of the PEDOT:PSS composite films following the spin-coating and subsequent thermal annealing processes, particularly in the presence of GOPS and FS3100 additives. Nevertheless, increasing the quantities of GOPS and FS3100 in the PEDOT:PSS composite films reduced their electrical conductivities (Figure 3b). While the initial electrical conductivity of **P** registers at 1.0 S cm^–1^, it becomes remarkably enhanced to 820 S cm^–1^ upon the incorporation of 5 wt % DMSO, denoted as **PD**. This significant improvement in conductivity can be attributed to phase separation, with alterations in the transformational morphology and conformation of the PEDOT:PSS film. However, the introduction of GOPS or FS3100 additives had a pronounced adverse effect on the electrical conductivity. For instance, when GOPS (1 wt %) was added to **F0G1** and FS3100 (10 wt %) was added to **F10G0**, their respective average electrical conductivities reached 479 and 193 S cm^–1^; **F10G0.5** and **F10G1** exhibited average electrical conductivities of 103 and 93 S cm^–1^, respectively. In contrast, the conductivities of **F20G1** and **F30G1** became unmeasurable, denoted as “N/A”, indicating that they surpassed the detection limit of the commercial four-point probe system.

To analyze the composition of the PEDOT:PSS composite films incorporating FS3100, Raman spectroscopy was applied to pristine FS3100, pristine PEDOT:PSS, and **F10G1** following laser excitation at a wavelength of 532 nm (Figure S1). The Raman spectra revealed distinctive features of each component of the PEDOT:PSS composite film. Specifically, the Raman signals from the PEO and polytetrafluoroethylene (PTFE) components originate from FS3100, representing the C–H stretching of PEO (indicated by the open circle markers at 1,093 and 2892 cm^–1^) and PTFE (indicated by the close circle markers at 557 and 1,297 cm^–1^), respectively. Furthermore, the Raman signals from PEDOT:PSS were characterized by the vibrational modes of PEDOT (denoted by rhombus markers) at 1524, 1452, 1383, and 1272 cm^–1^, corresponding to the asymmetric C_α_=C_β_, symmetric C_α_=C_β_, C_β_–C_β_, and C_α_–C_α^′^_ vibrations, respectively. Meanwhile, the vibrational modes of PSS (indicated by triangle markers) were observed at 445, 575, 988, 1124, and 1562 cm^–1^. As expected, the characteristic signals in the spectra of **F10G1** were in agreement with the respective compositions of FS3100 and PEDOT:PSS within the composite films.

### 3.3 Phase Separation Study of PEDOT:PSS Composite Films

**Figure 4:**
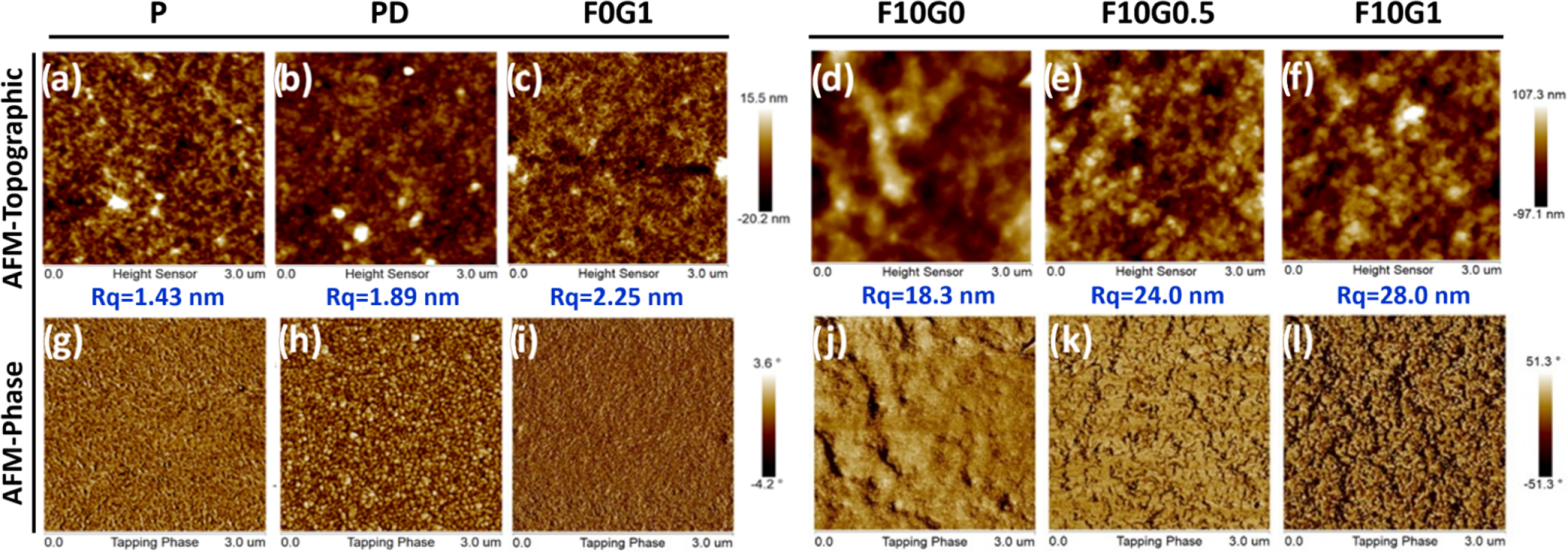
(a–f) Topographic and (g–l) phase AFM images of PEDOT:PSS composite films with different DMSO, GOPS, and FS3100 additive contents: (a, g) **P**, (b, h) **PD**, (c, i) **F0G1**, (d, j) **F10G0**, (e, k) **F10G0.5**, and (f, l) **F10G1** samples, obtained through tapping-mode AFM.

AFM was used in tapping mode to explore the nanophase separation within the PEDOT:PSS composite films in the presence of DMSO, GOPS, and FS3100 additives (Figure 4). This approach acquired topographic and phase images of the different films, facilitating the identification of PEDOT-rich and PSS-rich domains following the subsequent spin-coating and thermal cross-linking processes. As shown in Figure 4a–c, introducing both DMSO and GOPS did not significantly alter the surface root-mean-square roughness (Rq), as evident in the AFM topographic images with values of 1.43, 1.89, and 2.25 nm for **P**, **PD**, and **F0G1**, respectively. In the AFM phase images (Figure 4g–i), the **PD** film (with the addition of DMSO to **P**) exhibited a notable increase in the phase separation of continuous networks of the PEDOT-rich domains (depicted as bright regions) and PSS-rich domains (depicted as dark regions) when compared to that of the pristine **P** film. This is consistent with the findings of a prior study.^55^ However, the **F0G1** film (with the subsequent addition of GOPS to **PD**) impeded the phase-separation process. Notably, as the FS3100 content increased, the formation of fibrils was triggered, as previously documented.^57^ This film-fabrication process gives rise to larger continuous phase separations of PEDOT:PSS, as depicted in Figure 4j–l, and leads to a corresponding increase in surface roughness, with Rq values ranging from 18.3 to 28 nm (Figure 4d–f) within the PEDOT:PPS composite films. Therefore, the FS3100 additive was identified as capable of serving as a template, effectively constraining the phase separation of PEDOT:PSS into a continuous network. This phenomenon is pivotal in the development of highly electrically conductivity stretchable networks within **F10G0**, **F10G0.5**, and **F10G1**.^58^ To visually illustrate the continuous network formation within the PEDOT:PPS composite films, the phase images from Figure 4j–l were transformed into simplified black-and-white representations using specialized image processing software by ImageJ (Figure S2).^59^ The analyses revealed that the black area represents the PEDOT-rich domains, illustrating distinct continuous networks of PEDOT:PSS in both **F10G0** and **F10G0.5**. In Figure S2d, **F10G0** exhibited prominently large PEDOT-rich domains with a relatively low network density, while in Figure S2e, **F10G0.5** showcased smaller PEDOT-rich domains alongside a comparatively higher network density. Additionally, the **F10G1** film, depicted in Figure S2f, displayed a further reduction in the size of PEDOT-rich domains, accompanied by nonconductive gaps between these domains.

**Figure 5:**
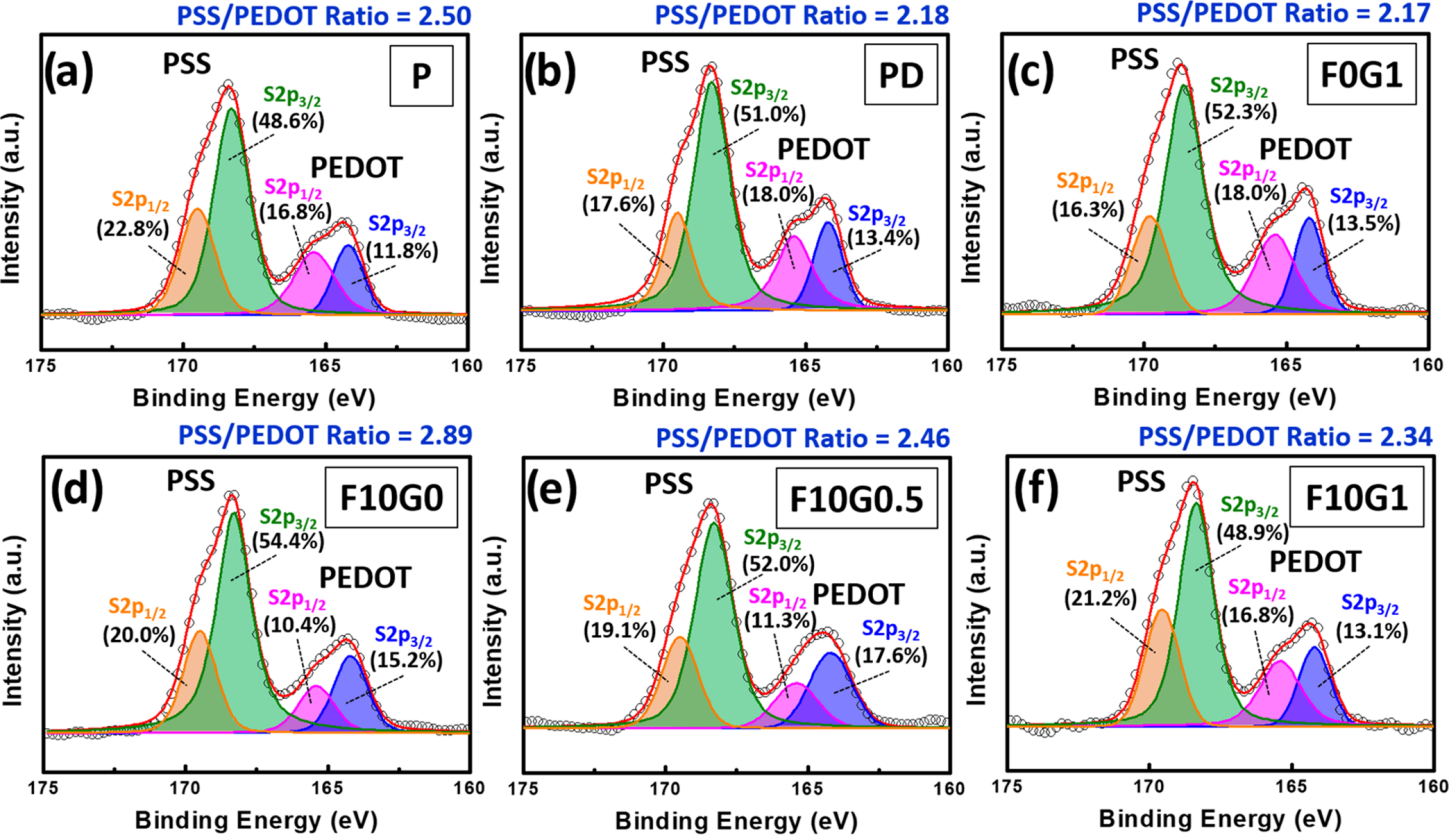
XPS S_2p_ spectra of PEDOT:PSS composite films with different DMSO, GOPS, and FS3100 additive contents: (a) **P**, (b) **PD**, (c) **F0G1**, (d) **F10G0**, (e) **F10G0.5**, and (f) **F10G1** samples.

In addition to observing the morphological changes resulting from phase separation, high-resolution XPS core-level spectra (S_2p_) were used to assess alterations in the surface composition of the PSS/PEDOT ratio following the incorporation of DMSO, GOPS, and FS3100 into the **P** films (Figure 5). Consequently, the incorporation of 5 wt % DMSO into the PEDOT:PSS dispersion led to a notable alteration in the PSS/PEDOT ratio within the composite film, shifting it from 2.50 (referred to as **P**) to 2.18 (referred to as **PD**) (Figure 5a,b), which is similar to previously reported value.^59^ Furthermore, the addition of 1 wt % GOPS to the composite film resulted in only a slight decrease in the PSS/PEDOT ratio, from 2.18 (referred to as **PD**) to 2.17 (referred to as **F0G1**) (Figure 5b,c). The calculated surface energies for the PTFE and PEO chains derived from FS3100 were 24.41 and 38.48 mJ m^–2^, respectively (Figure 1). These values are notably lower than those for PSS (γ = 54.1 mJ m^–2^), PEDOT (γ = 50.6 mJ m^–2^), and PSS-GOPS (γ = 73.8 mJ m^–2^). The difference in surface energies may explain the observed phase separation during film formation when the PSS/PEDOT ratio changed from 2.18 (referred to as **PD**) to 2.89 (referred to as **F10G0**). In contrast, the PSS/PEDOT ratio for **F0G1** remained almost constant, at approximately 2.17, owing to the limited addition of GOPS (1 wt %) to **F0G1**.

**Figure 6:**
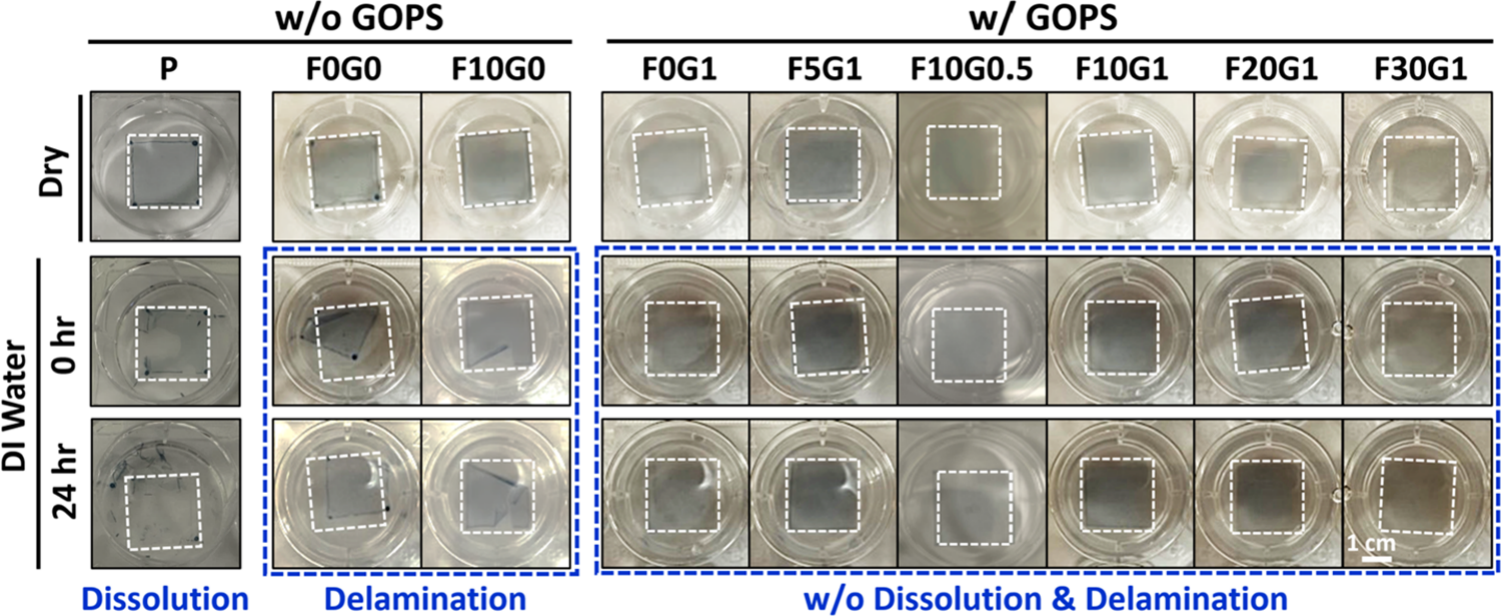
Photographs and water resistance tests of PEDOT:PSS composite films with different DMSO, GOPS, and FS3100 additive contents.

To verify the water stability of the cross-linked structures formed within the PEDOT:PSS films owing to the phase separation induced by the presence of DMSO, FS3100, and GOPS additives on the glass substrates, a water resistance test was performed in DI water over 24 h to assess the ability of the films to withstand dissolution and delamination (Figure 6). The dashed square lines in Figure 6 represent individual glass substrates with different PEDOT:PSS composite film coatings. The **F0G0** film is the same as the **PD** film shown in Figure 2. Notably, the as-prepared **PD** and **F10G0** films (without GOPS addition) effectively prevented the dissolution of the PEDOT:PSS films but exhibited some film delamination. Therefore, obtaining the ζ-potential of the **PD** film through EKA measurements under external pressure supply conditions was unfeasible. However, as well as acting as the thermal cross-linker in all of the composite films and eliminating the dissolution problem, the additional incorporation of 0.5–1 wt % GOPS within the PEDOT:PSS films also improved adhesion performance on the glass substrate, effectively eliminating the delamination issue while maintaining good water resistance properties.^50^

To gain deeper insights into the transition from a PEDOT:PSS composite solution to a solid-state film, we conducted additional experiments. These included assessments of dynamic viscosity, water contact angle (WCA), and ζ-potential measurements for **F10G0**, **F10G0.5**, **F10G1**, and **F10G5** composite materials. These experiments aimed to comprehend alterations in the viscosity of solutions, WCA of films, ζ-potential of films, and surface composition during the spin-coating process, coinciding with the vertical phase separations of FS3100, PEDOT:PSS, and PSS-GOPS domains within the PEDOT:PSS composite films. Initially, to explore the impact of GOPS on PEDOT:PSS composite materials enhanced with 10 wt % FS3100 additives (**F10GX**, X ranging from 0 to 0.5, 1, and 5 wt %), dynamic viscosity measurements were conducted for elucidating viscosity changes. These measurements aimed to unveil shifts in viscosity, revealing a progressive increase with values of approximately 589, 600, 603, and 615 cP for **F10G0**, **F10G0.5**, **F10G1**, and **F10G5**, respectively, measured at a shear rate of approximately 10 s^–1^ (Figure S3a). It was anticipated that varying concentrations of PSS-GOPS domains in **F10GX** solutions would minimally impact the hydrogen-bonded networks, resulting in marginal viscosity changes. However, the examination unveiled a significant reduction in water contact angles (WCAs): approximately 19.9° ± 0.6°, 12.4° ± 0.3°, 8.3° ± 0.5°, and 2.2° ± 0.3° for **F10G0**, **F10G0.5**, **F10G1**, and **F10G5** films, respectively (Figure S3b). This transition from a solution to a solid-state film highlights a noteworthy phenomenon: as the GOPS concentration exceeds 1 wt %, PSS-GOPS-rich domains develop on the surface of **F10GX** films, leading to the creation of superhydrophilic surfaces (WCA < 5°). This discovery aligns with the wettability findings outlined in a previous report.^54^

Furthermore, in the fabrication of **F10GX** films through the spin-coating process at 4000 rpm for 60 s, the ζ-potentials exhibited an increasing trend in negative charge. They measured −12.6 ± 0.2 mV for **F10G0**, −18.4 ± 1.0 mV for **F10G0.5**, −31.0 ± 0.2 mV for **F10G1**, and −40.3 ± 0.7 mV for **F10G5**. This trend signifies that the higher presence of PSS-GOPS domains (δ = 31.2 J^1/2^/cm^3/2^; γ = 73.8 mJ m^–2^) within **F10GX** films favors a vertical phase separation of PSS-rich domain as the solvent evaporates during the spin-coating, resulting in an increased negative charge (Figure S3c). However, in the case of **F10G0.5** films, the ζ-potentials decreased from −18.4 ± 1.0 mV (at 4000 rpm) to −9.9 ± 0.9 mV (at 3000 rpm) and further to −8.1 ± 0.6 mV (at 2000 rpm) (Figure S3d). This divergence occurs due to the similar solubility parameters between the DMSO solvent (δ = 26.6 J^1/2^/cm^3/2^) and PSS (δ = 24.9 J^1/2^/cm^3/2^). At lower spin-coating speeds, the slower evaporation of DMSO as a solvent reduces the driving forces that encourage the vertical phase separation of PSS-rich domains toward the outer layer. Consequently, this leads to lower ζ-potential values indicating a decrease in negative charge. Moreover, Figure S4 illustrates the distinctive in-depth profiles of the **F10G0.5** film. The C^–^, 18O^–^, F^–^, S^–^, and Si^–^ profiles offer precise insights into the vertical phase separation among the FS3100, PEDOT, PSS, and PSS-GOPS domains. As anticipated, the outer layer predominantly comprises FS3100-rich domains [inclusive of PTFE (γ = 24.4 mJ m^–2^) and PEO (γ = 38.5 mJ m^–2^)], evident from the notably heightened C^–^ and F^–^ signals compared to those of other selected ions. The inner layer consists primarily of PEDOT:PSS [comprising PEDOT (γ = 50.6 mJ m^–2^) and PSS (γ = 54.1 mJ m^–2^)] alongside PSS-GOPS domains (γ = 73.8 mJ m^–2^), highlighted by the intensified S^–^ and Si^–^ signals that reach their maximum intensity at a depth of approximately 100 nm within the **F10G0.5** film.

### 3.4 Morphological Model of PEDOT:PSS Composite Solutions and Films

**Figure 7:**
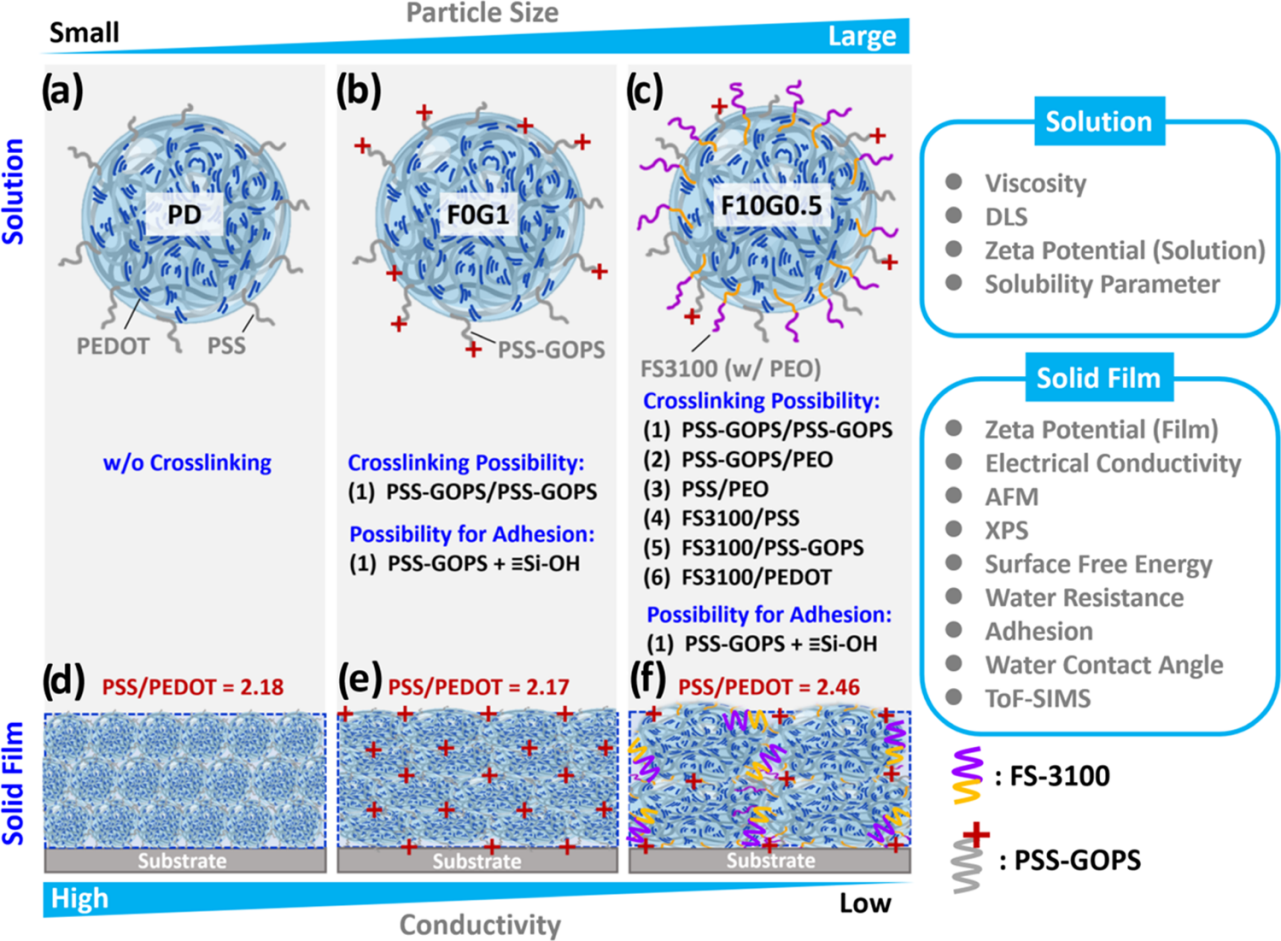
Schematic representations of morphological models depicting (a–c) PEDOT:PSS-based colloid solutions and their resulting (d–f) composite solid films with different DMSO, GOPS, and FS3100 additives, featuring the (a, d) **PD**, (b, e) **F0G1**, and (c, f) **F10G0.5** samples.

Based on a comprehensive analysis of the calculated surface energies, viscosities, size distributions, ζ-potentials, electrical conductivities, AFM images, and XPS results of the PEDOT:PSS composite materials, Figure 7 shows correlated morphological models to elucidate the behavior of the PEDOT:PSS composite films in the presence of DMSO, GOPS, and FS3100. This aids in comprehending the phase separation within the PEDOT:PSS films, effectively bridging the explanatory gaps from the molecular level to macroscopic morphological changes. For instance, first, several PSS on the surface of **F10G0.5** composite colloids provide the possibility of forming a dynamic cross-linking process involving FS3100/PSS through hydrogen bonding and, thereby, an enlargement in the particle size of PEDOT:PSS colloids compared to **PD** and **F0G1** (Figure 7a–c). Second, within **F10G0.5**, potential cross-linking mechanisms were identified including three thermal cross-linking pathways involving the covalent bonding of PSS-GOPS/PSS-GOPS, PSS-GOPS/PEO, and PSS/PEO interactions, as well as three dynamic cross-linking processes involving FS3100/PSS and FS3100/PSS-GOPS through hydrogen bonding and FS3100/PEDOT via electronegative fluorine bonding (Figure S5). These cross-linking phenomena are believed to enhance water resistance and contribute significantly to self-healing. Third, PSS-GOPS functioned as an adhesion promoter in the **F0G1** and **F10G0.5** films, effectively preventing the delamination of the hydroxyl-modified substrates (Figure 7d–f). Fourth, although the inclusion of GOPS and FS3100 led to a decreased electrical conductivity, it also significantly enhanced the continuous phase separation between the PEDOT-rich and PSS-rich domains compared to that of the **PD** and **F0G1** films. The enhancement in phase separation is thought to play a pivotal role in establishing continuous networks within the PEDOT-rich domains, thereby enhancing the stretchability of PEDOT:PSS composite films.^60^ This advancement renders them well-suited for use in wearable electronics.

### 3.5 Self-Healing Capabilities of PEDOT:PSS Composite Films

**Figure 8:**
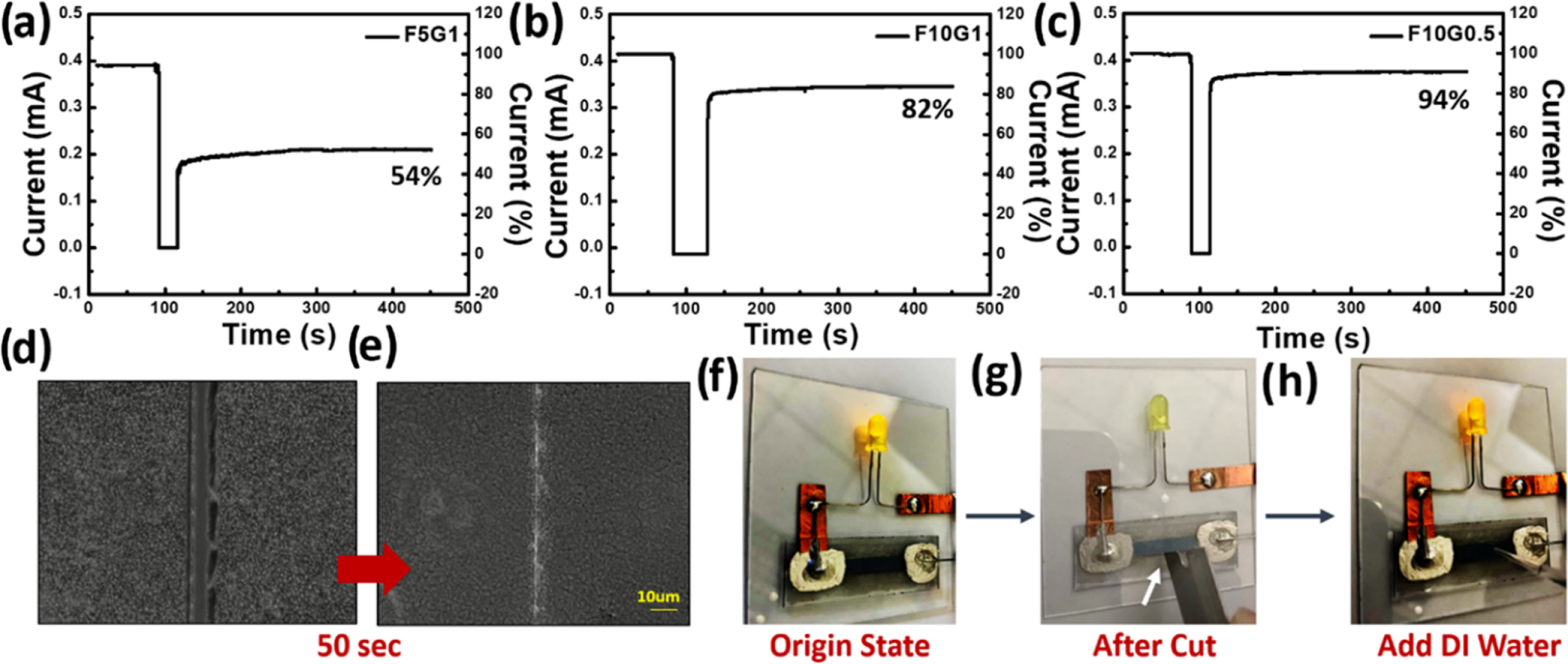
Self-healing properties of PEDOT:PSS composite films. Current versus time profiles of (a) **F5G1**, (b) **F10G1**, and (c) **F10G0.5** films, with an approximately 10 μm width gap cut by a blade before and after a DI water drop was placed on the gap area. The applied voltage was 0.2 V. (d) Optical images of the **F10G0.5** film with the damaged area (d) before and (e) after the addition of the DI drop and drying at room temperature. (f–h) Images showing the damaged area and self-healing ability of an **F10G0.5** film connected to a circuit with an LED bulb at a constant voltage of 1 V: (f) as-prepared; (g) film damage with a cut; and (h) film healed by dropping DI water on the cut area.

Based on the previously discussed morphological models that elucidate the behavior of PEDOT:PSS composite films in the presence of DMSO, GOPS, and NIFS additives, the higher proportion of PSS/PEO and PSS/PTFE dynamic cross-linking reactions through hydrogen bonding within the films likely significantly enhances their self-healing capabilities. As a result, **F10G0.5** offers optimized electrical conductivity, self-healing performance, and film adhesion (Figure 8). One compelling reason for this assumption is that **F10G0.5** yields a PSS/PEDOT ratio that is higher than that of **F10G1**. Furthermore, with the incorporation of 10 wt % FS3100 by **F10G0.5**, there will be an excess of PTFE chains, facilitating the formation of additional PSS/PTFE interactions through OH···F hydrogen bonding, especially when contrasted with **F5G1**. Consequently, just 0.5 wt % GOPS in **F10G0.5** should be sufficient to achieve superior adhesion to the glass substrate in the resulting composite film. To validate this, three distinct formulations, **F5G1**, **F10G1**, and **F10G0.5**, were prepared, and their correlated self-healing capabilities were investigated. This involved the real-time monitoring of the current response from the initial state to single-line cutting-induced breakage and, last, the water-assisted self-healing phase (Figure 8a–c). Based on this, **F10G0.5** exhibited an impressive current recovery rate of 94%, significantly surpassing that of **F10G1** (82%) and **F5G1** (54%). The optical microscope images of the healed films (Figure 8d–e) reveal that while the damage caused by the blade remained discernible, there were no longer any discontinuities between the two sides of the cut after 50 s of water-assisted self-healing. As previously referenced,^41^ the utilization of pressure contact across various physical applications is believed to offer a promising approach in substantially augmenting the recovery rate of current signals within our established PEDOT:PSS composite film system. Effectively, appropriate pressure contact can expedite the healing process of the impaired regions upon their reconnection. Consequently, future investigations aim to improve the deformability and softness of the PEDOT:PSS composite film, thereby enhancing its ability to better repair cracks on polymer films.

The self-healing capabilities of the **F10G0.5** film were further demonstrated by intentionally damaging and repairing it through connection to a primary circuit with a commercial light-emitting diode (LED) light bulb (Figure 8f–h). Notably, the LED was deactivated when the film was cut but promptly reactivated when it was repaired using DI water. Based on our established morphological models (Figure 7), this healing effect is tentatively ascribed to the swelling of excess PSS and/or PSS-GOPS chains upon exposure to water^51^ and the dynamic cross-linking processes through hydrogen bonding. For example, swelling results in an increased phase separation and film softening. Additionally, the high surface energy of PSS-GOPS was exposed after cutting-induced breakage. Simultaneously, the lower surface energies of the PSS-rich and FS3100 domains tended to migrate to the edges of the film, facilitating the healing of the damaged structures through a dynamic cross-linking process involving hydrogen bonding.^61^ These hydrogen bonds induce decohesion within the PEDOT:PSS composite colloids, increasing the mobility of PSS-rich domains in the DI water. Consequently, this accelerates the separation and migration of PSS-rich domains to the damaged area. Subsequently, as water evaporates, the previously broken hydrogen bonds reform, restoring the cohesion between the grains. The healing mechanism involves dynamic hydrogen bonding, allowing for the high swelling ability of **F10G0.5** composite materials in this study, a process regulated by the presence of water. Prior research has demonstrated that GOPS molecules can form cross-links with PSS as well as other GOPS molecules and glass substrates, thereby improving the mechanical properties of PEDOT:PSS films. Additionally, the compound containing multiple hydroxyl groups suggests a potential enhancement in both the mechanical properties and the self-healing capability of the PEDOT:PSS composite films. This is attributed to the anticipated influence of FS3100, which aids in the vertical phase separation, allowing for the migration of negatively charged PSS to the surface of the PEDOT:PSS composite films during the spin-coating process.^62,63^

### 3.6 Flexibility Tests of LSG and PEDOT:PSS Films on PDMS Substrates

**Figure 9:**
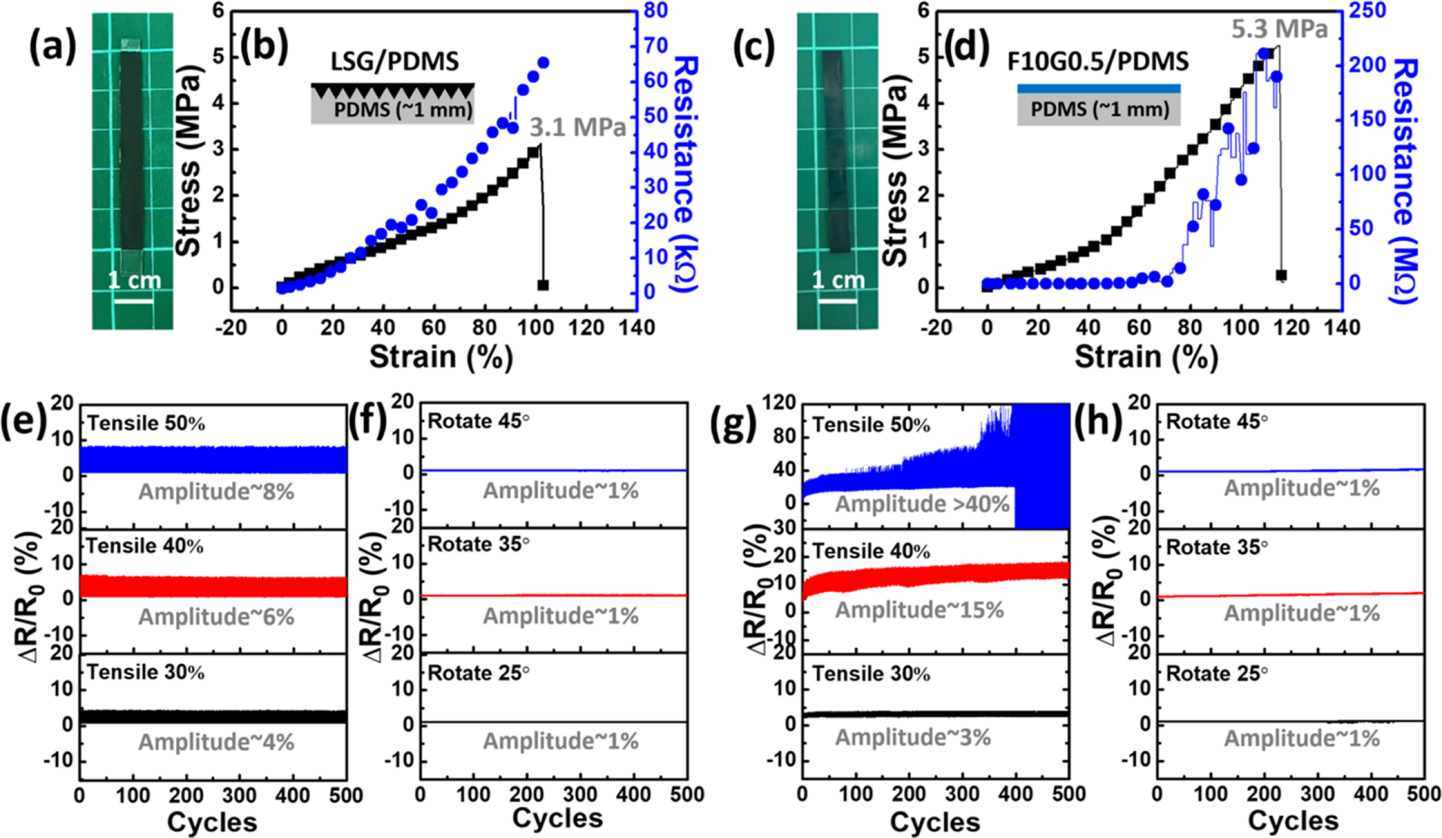
Evaluation of the mechanical strength and associated resistance changes of wearable **F10G0.5**-based OECTs. Photographs of (a) **LSG/PDMS** and (c) **F10G0.5/PDMS** samples (50 mm × 5 mm) and their (b) tensile and (d) resistance tests. Long-term stability tests for LSG/PDM and **F10G0.5** films under (e, g) tensile (strain from 4 to 5 and 8%) and (f, h) rotation (twisted angle from 25 to 35 and 45°) tests, respectively.

Figure S6a,b illustrates the SEM images of three-dimensional **LSG**, displaying a porous morphology characterized by isotropic pores and sheet-like structures. Additionally, nanofibers are observed atop these isotropic pores and sheet-like structures. Analysis of the Raman spectrum of the **LSG** electrode unveiled an *I*_D_/*I*_G_ ratio of 0.90, indicating the successful creation of well-defined graphene domains through the CO_2_ laser scribing technology (Figure S6c). This finding aligns with a previous literature report.^52^ In addition, the sheet resistances of **LSG** and **LSG/PDMS** electrodes were approximately 80.4 and 109.3 Ω/sq., respectively (Figure S6d). To satisfy the mechanical properties required for the development of wearable PEDOT:PSS-based OECTs on PDMS substrates, stress–strain (SS), cyclic tensile, and cyclic twist tests were performed on **LSG** layers and **F10G0.5** films affixed to the PDMS substrates, referred to as **LSG/PDMS** and **F10G0.5/PDMS**, respectively (Figure 9). For the SS tests, all **LSG/PDMS** and **F10G0.5/PDMS** specimens were prepared using the PDMS transfer and spin-coating processes and then cut using a single-edge razor blade (width = 0.5 cm, length = 4 cm, thickness = 1 mm), as shown in Figure 9a,c, respectively. The stress–strain relationships were measured by analyzing the mechanical strength and fracture load differences until failure as well as the corresponding resistance responses (Figure 9b,d). A 1 mm thick **LSG/PDMS** specimen demonstrated a mechanical strength of 3.1 MPa with approximately 103% elongation at failure and, moreover, exhibited a linear increase in resistance from 1.37 to 65.5 kΩ. Conversely, the 1 mm thick **F10G0.5/PDMS** specimen exhibited a mechanical strength of 5.3 MPa with an elongation at failure of approximately 115% and a resistance increment that followed an exponential pattern, with a turn-on strain of 70% and a range of 1.64–211.0 MΩ. As the application of tensile strain is recognized to induce crack formation across the thickness of the PEDOT:PSS film on stretchable PDMS substrates,^64^ the observed fluctuation in the resistance response curve of **F10G0.5/PDMS** could reasonably be attributed to the self-healing mechanism on addressing cracks, particularly noticeable when the strain surpassed 70%. Meanwhile, PDMS demonstrated a more uniform mechanical behavior, as reflected in the minimal fluctuation of its stress response in the context of **F10G0.5/PDMS** on the PDMS substrate (Figure 9d). The relative resistance change (Δ*R*/*R*_0_) in the **LSG/PDMS** specimens showed a modest increase, progressing from 4 to 6 and 8% during cyclic tensile testing, with tensile strains increasing from 30 to 40 and 50%, respectively. Additionally, the increase was minimal (approximately 1%) when subjected to twist testing at rotation angles of 25, 35, and 45°. Conversely, the relative resistance change (Δ*R*/*R*_0_) in the **F10G0.5/PDMS** specimens showed a gradual escalation, ranging from 3 to 15% and exceeding 40% (leading to failure after 400 cycles) during cyclic tensile testing, with tensile strains of 30 to 40 and 50%, respectively. Notably, the increase in the relative resistance change (Δ*R*/*R*_0_) remained minimal, at approximately ∼1%, when the specimens are subjected to twist testing at rotation angles of 25, 35, and 45°. Therefore, these preliminary results demonstrate that the developed **LSG** and **F10G0.5** coatings on PDMS substrates offer excellent flexibility, making them suitable for use as source/drain electrodes and active-layer channels in potential wearable OECT devices.

### 3.7 Device Characterization of Wearable OECTs

**Figure 10:**
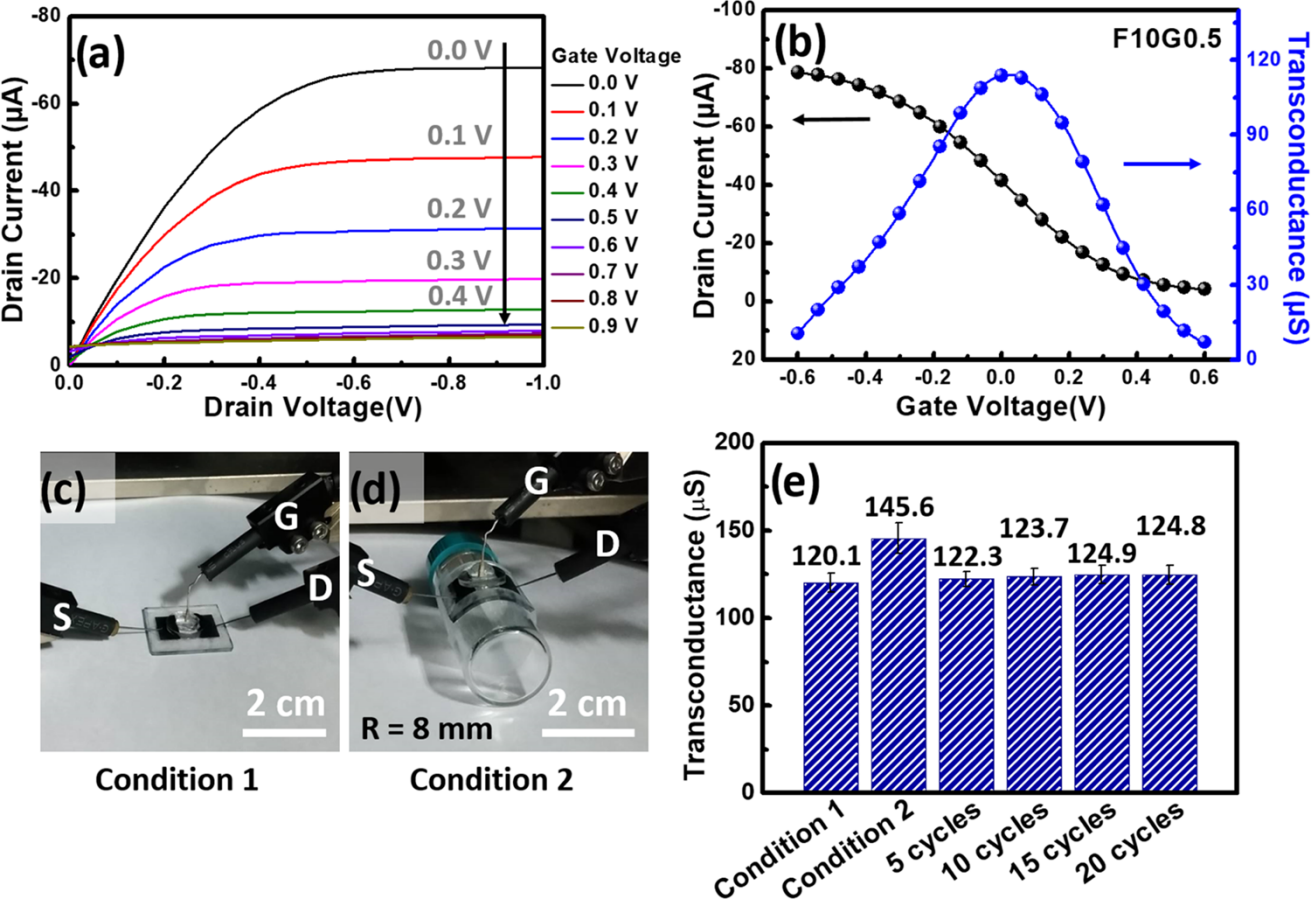
Output characteristics for **F10G0.5**-based OECTs. (a) Output (*I*_d_–*V*_d_) and (b) transfer characteristics (*I*_d_–*V*_g_) and the associated transconductance (*g*_m_) peak curves of OECTs. Photographs of OECTs (c) before bending and (d) after bending (*R* = 8 mm) on a flexible PDMS substrate. (e) Associated *g*_m_ for OECTs after multiple cyclic bending tests.

To demonstrate the integration of **F10G0.5/PDMS** as the active-layer channel and **LSG/PDMS** as the source and drain electrodes in wearable OECTs designed for biosensing applications, several key measurements were obtained in PBS buffer (1×) at pH 7.4. The detailed fabrication process of the wearable **F10G0.5**-based OECTs on the **LSG/PDMS** substrate is illustrated in Figure S7. This process used CO_2_ laser scribing technology to generate two patterned LSG electrodes that served as the source and drain electrodes. Additionally, a PDMS transfer process was used to obtain the **LSG/PDMS** substrate, spin-coating, and CO_2_ laser patterning processes of the **F10G0.5** composite solution to create the active-layer channel for the OECTs and the encapsulation of a PDMS chamber to create a biosensing device for DA detection. The measurements included the drain current–drain voltage (*I*_d_–*V*_d_) output characteristics, drain current–gate voltage (*I*_d_–*V*_g_) transfer curve, and the corresponding transconductance (*g*_m_)–*V*_g_ curve. Furthermore, the alterations in the *g*_m_ value were monitored over multiple cycles of bending tests, providing valuable insights into device stability (Figure 10). Figure 10a,10b shows the output characteristics of the **F10G0.5**-based OECT. These measurements were taken with a negative sweeping bias, ranging from 0 to −1.0 V on the drain, while the gate voltages varied from 0 to 0.9 V. The experimental setup involved inserting a Ag/AgCl gate electrode into the buffer solution. The results revealed that the OECT based on **F10G0.5/PDMS** and **LSG/PDMS** exhibited typical transistor behavior. In particular, the capability of modulating the drain current as the applied gate voltage increases was well demonstrated, which achieved a maximum *g*_m_ value of 114 μS at a gate voltage of 0.04 V. The OECT device maintained a stable performance during bending tests with a fixed bending radius (*R*) of 8 mm (Figure 10c–e). The corresponding peak transconductance exhibited minimal variation (<1%) even after multiple bending cycles (e.g., 5, 10, 15, and 20 cycles), emphasizing its robustness in the presence of physical deformation. Additionally, a slight increase in transconductance under the bending test (condition 2) was attributed to the expanded sensing area of the active-layer channel after stretching.

An extended long-term stability test was carried out on the **F10G0.5**-based OECTs (Figure S8). Impressively, these OECTs showcased exceptional “spike and recovery” current changes (Δ*I*) of 48.9 μA, exhibiting only a marginal decrease of 98.9% over 500 cycles of the OECT operation. This underscores their remarkable device stability when compared to the cross-linked PEDOT:PSS-based OECTs.^65^

### 3.8 Wearable OECTs for Biosensing Applications

**Figure 11:**
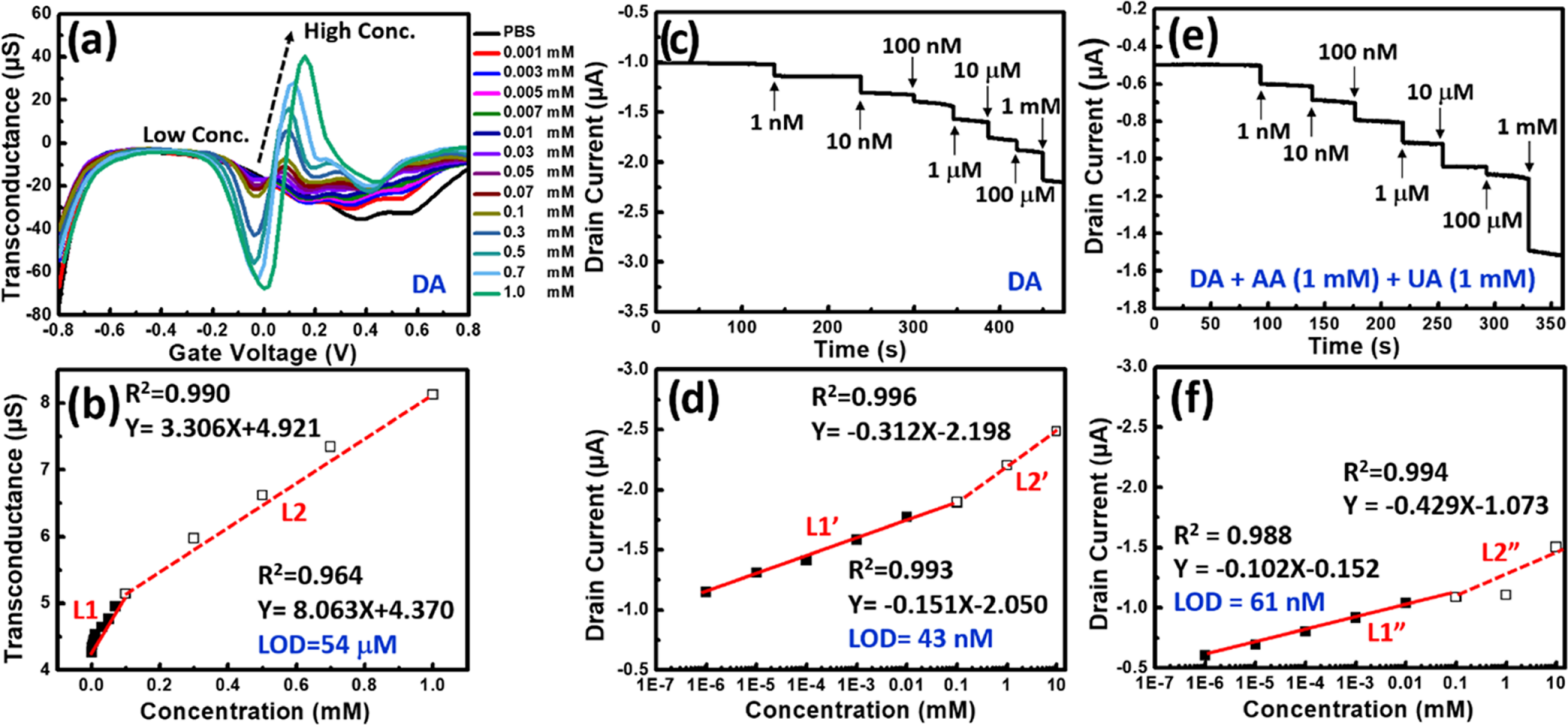
**F10G0.5**-based OECTs for detecting DA. (a) Transconductance response curves and (b) correlated calibration curves of OECTs and recorded in 1× PBS (pH 7.4) containing DA (1 μM–1 mM) with a *V*_d_ of 0.1 V: gate potential *V*g swept from −0.8 to +0.8 V. (c) Amperometric response curves (*I*_d_-time) and (d) correlated calibration curves of OECTs recorded with incremental additions of DA (1 nM–1 mM). (e) Amperometric response curves (*I*_d_-time) and (f) correlated calibration curves of OECTs recorded with incremental additions of DA (1 nM–1 mM) in the presence of AA (1 mM) and UA (1 mM).

Because OECTs can transform ionic signals originating from biological sources into electronic signals, their effectiveness can be evaluated by measuring their *g*_m_ value. The transconductance signals were extracted from the *I*_d_–*V*_g_ transfer curve, which featured a characteristic peak corresponding to the gate voltage. Therefore, this shifted transconductance peak was highly valuable for monitoring changes in the concentration of redox chemicals, making it an invaluable asset for biosensing applications. In this study, **F10G0.5**-based OECTs were used to demonstrate their performance in detecting DA (Figure 11). As shown in Figure 11a–b, the characteristic transconductance peaks of the OECTs appeared as the gate voltage increased from 0.04 to 0.16 V in response to increasing DA concentrations, ranging from 0 to 1.0 mM, within a 1× PBS (pH 7.4) buffer, at a constant *V*_d_ of 0.1 V. This observation underscores the strong linear performance of the developed wearable OECT devices [*g*_m(DA)_ = 8.063*C*_DA_ + 4.370, *R*^2^ = 0.964], characterized by an LOD (S/N = 3) of 54 μM for low DA concentrations in the range of 1–100 μM. Moreover, for higher concentrations of DA (100–1000 μM), the OECT device still demonstrated good linear performance [*g*_m(DA)_ = 3.306*C*_DA_ + 4.921, *R*^2^ = 0.990].

After identifying the characteristic transconductance peaks of DA based on previous measurements of the transconductance response curves (Figure 11a,b), an additional measurement was made to assess the *I*_ds_ response across various DA concentration constant *V*_d_ and *V*_g_ values of 0.1 V (Figure 11c,d). When studying the *I*_d_-time transfer curves, this approach yielded a superior linear performance and a lower LOD. For instance, in cases where higher degrees of DA oxidation occurred within the **F10G0.5**-based OECT, there was greater consumption of cationic species, leading to an increase in the negative *I*_d_ values (Figure 11c). This sensing behavior was characterized by two empirical equations (Figure 11d)—a linear regression equation for low DA concentrations: (1 nM–100 μM) of *I*_DA_ = −0.151*C*_DA_–2.050 (*R*^2^ = 0.993) with an LOD of 43 nM; and a corresponding linear regression equation for higher DA concentrations (100–1000 μM): *I*_DA_ = −0.312*C*_DA_–2.198 (*R*^2^ = 0.996).

Finally, the **F10G0.5**-based OECT was employed to assess its sensing performance for the highly selective determination of DA in the presence of AA and UA as potential interfering agents (Figure 11e,f). Figure 11e illustrates the *I*_d_-time transfer curves recorded at various DA concentrations (1 nM to 100 μM) in a 1× PBS (pH 7.4) buffer, with AA and UA present. This sensing behavior was further described by two empirical equations (Figure 11f)—a linear regression equation for lower DA concentrations (1 nM–100 μM): *I*_DA_ = −0.102*C*_DA_–0.152 (*R*^2^ = 0.988), featuring a LOD of 61 nM, which was slightly higher than that observed under DA-only condition (Figure 11d); and for higher DA concentrations (100–1000 μM): *I*_DA_ = −0.429*C*_DA_–1.073 (*R*^2^ = 0.994), demonstrating similar detection performance as the DA-only condition (Figure 11c,d). Microdialysis and fast-scan cyclic voltammetry (FSCV) are widely recognized methods for measuring DA concentration.^66^ Microdialysis stands out for its exceptional sensitivity, allowing for the collection of samples that can be subsequently separated and analyzed by using high-performance liquid chromatography and mass spectrometry. In contrast, FSCV, also known as differential pulse voltammetry (DPV), is a cost-effective method that offers outstanding temporal and spatial resolutions. It achieves subsecond resolution for mapping DA release events over time. Furthermore, as demonstrated in a previous study on PEDOT:PSS-based OECT devices,^32^ OECT sensors exhibit superior sensitivity and the lowest LOD for DA detection compared to CV and DPV methods. Notably, our developed **F10G0.5**-based OECT, utilizing the *I*_d_-time transfer curve approach, achieves an LOD of 43 nM within the linear range of 1 nM to 100 μM. This LOD represents a significant improvement over the previous report, which noted an LOD of 6 μM within the linear range of 5–100 μM.

## 4 Conclusions

The additive blending effects of DMSO, GOPS, and a nonionic FS3100 fluorosurfactant in forming PEDOT:PSS composite films for use as active-layer channels in OECTs were systematically assessed. A morphological model for the PEDOT:PSS composite films was also proposed, offering a clear explanation for their exceptional properties, including high electrical conductivity, flexibility, stretchability, self-healing capabilities, and water resistance. Primarily, **F10G0.5** exhibited a more pronounced decrease in the PSS/PEDOT ratio than both **P** and **F10G0**. This can be attributed to the increased surface energy required for the formation of PSS-GOPS. Consequently, more PSS-GOPS leads to the migration of PSS from the outer surface toward the interior of the PEDOT:PSS composite films, ultimately facilitating the development of a fibrous network that contributes to the high flexibility, stretchability, and self-healing properties of the PEDOT:PSS composite films. The **F10G0.5** film exhibits an impressive current recovery rate of 94%, surpassing that of the **F10G1** film. This superior performance can be attributed to the presence of more PSS on the surface, which facilitates the formation of more dynamic cross-linking networks, thus enhancing its self-healing capability. Subsequently, optimized **F10G0.5/PDMS** was successfully integrated as the active-layer channel and **LSG/PDMS** as the source and drain electrodes into a wearable OECT device to demonstrate its biosensing applications. The flexible **F10G0.5**-based OECTs were employed in the electrochemical biosensing of DA in the presence of multiple interferents, including AA and UA, and provided an amperometric response to DA with an LOD of 61 nM in the linear range of 1 nM–100 μM. Overall, this study on the effects of additive blending and the resulting morphological film model holds the potential to advance the development of PEDOT:PSS composite films with excellent stretchability, twistability, and self-healing properties, thereby aligning them with the requirements of wearable biosensing applications.

## Data Availability

The data that has been used is confidential.

## Supporting Information Available

PDI, hydrodynamic radius, viscosities of PEDOT:PSS composite solutions; Raman spectra; AFM images; water contact angle; ζ-potential; ToF-SIMS depth profiles; potential mechanisms of PEDOT:PSS composite films; SEM images and Raman spectrum of LSG and LSG/PDMS electrodes; and the fabrication process and long-term (ID-time) stability assessments of wearable OECT devices (PDF)
