# Supplementary material for: Additive Blending Effects on PEDOT:PSS Composite Films for Wearable Organic Electrochemical Transistors
Source: ACS Appl Mater Interfaces. 2024 Mar 8;16(11):13384–98. doi: 10.1021/acsami.3c14961 (PMC10958448; doi:10.1021/acsami.3c14961)
Supplement: Supplementary file 1 — am3c14961_si_001.pdf [file am3c14961_si_001.pdf]

Supporting Information

Additive Blending Effects on PEDOT:PSS Composite Films for Wearable Organic Electrochemical Transistors

Hsueh-Sheng Tseng, Ying-Lin Chen, Pin-Yu Zhang, and Yu-Sheng Hsiao*

Department of Materials Science and Engineering, National Taiwan University of Science and Technology, Taipei, 10607, Taiwan

*Corresponding author at:

Prof. Yu-Sheng Hsiao: Department of Materials Science and Engineering, National Taiwan University of Science and Technology, Taipei 10607, Taiwan;

E-mail: yshsiao@mail.ntust.edu.tw

Table. S1 Polydispersity index (PDI) and hydrodynamic radius of PEDOT:PSS composite solution by DLS analysis.

Sample Name	Polydispersity Index (PDI)	Hydrodynamic Radius (nm)
**P**	0.27	445.8 ± 76.5
**PD**	0.28	353.2 ± 30.5
**F0G1**	0.26	351.9 ± 4.0
**F3G1**	0.29	282.5 ± 51.5
**F5G1**	0.25	251.2 ± 32.6
**F10G1**	1.16	7989.0 ± 979.2
**F10G0.5**	1.42	6034.1 ± 763.4
**F10G0**	0.85	7626.5 ± 3662.8

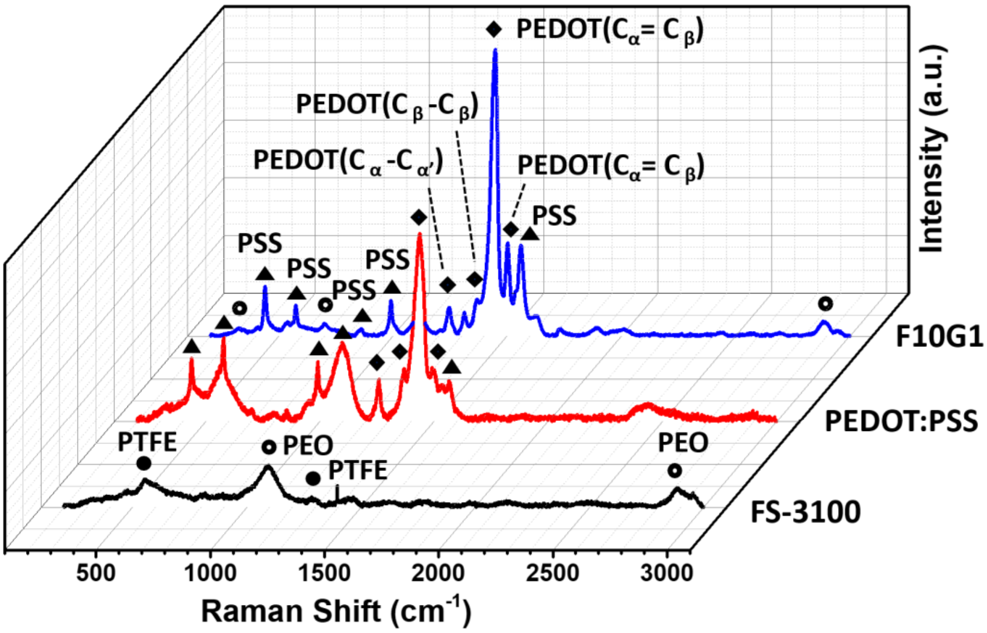

Figure S1. Raman spectra of pristine FS-3100, PEDOT:PSS, and **F10G1** films.

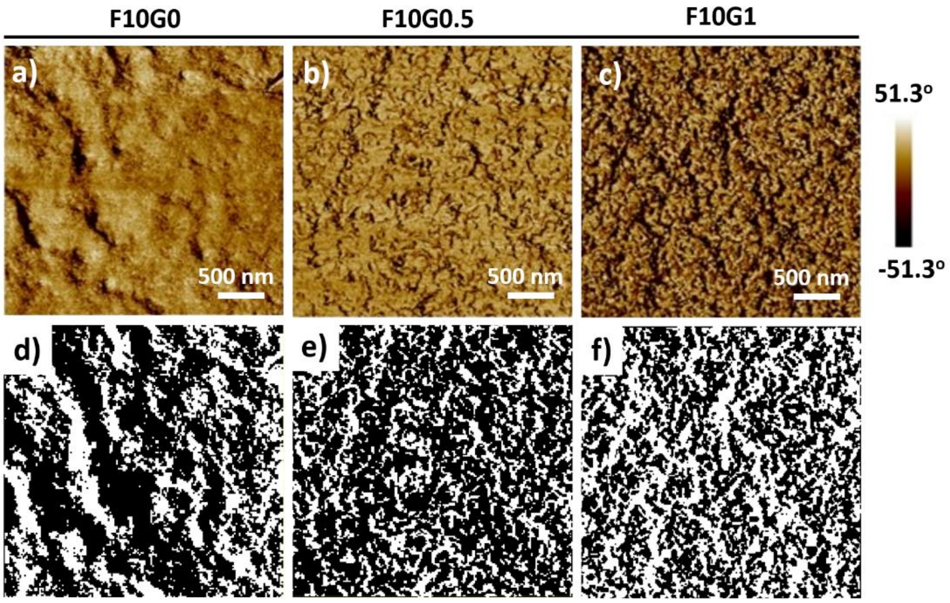

Figure S2. AFM phase images of PEDOT:PSS composite films, including (a) **F10G0**, (b) **F10G0.5**, and (c) **F10G1**. Image processing of original AFM phase images with the threshold of 20/255 for black-and-white representations of (d) **F10G0**, (e) **F10G0.5**, and (f) **F10G1**.

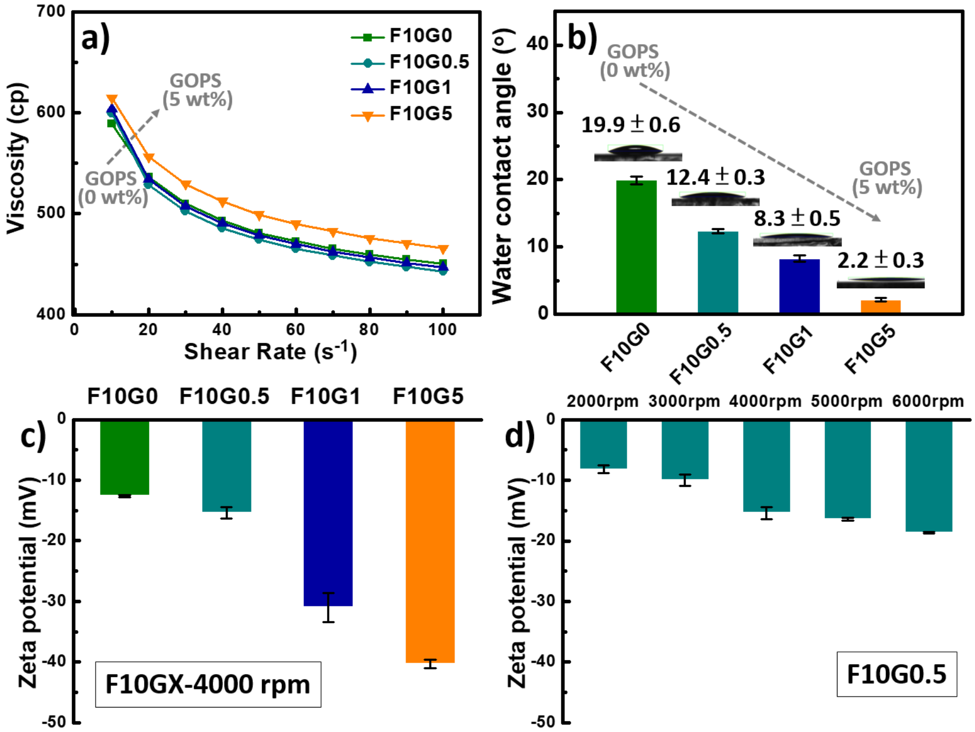

Figure S3. (a) viscosities, and (b) water contact angles of **F10GX** composite (**X** ranging from 0 to 0.5, 1, and 5 wt%) solutions and films, respectively. (c) Zeta potential of **F10GX** composite films. (d) Zeta potential of **F10G0.5** composite films as a function of spin-coating speeds (ranging from 2000 to 6000 rpm).

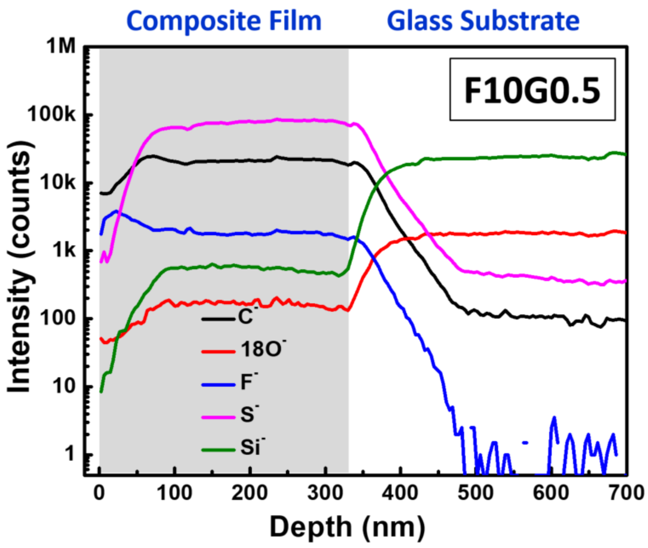

Figure S4. ToF-SIMS depth profiles of the **F10G0.5** composite film (fabricated at 2000 rpm for 60 s by using the spin-coating process).

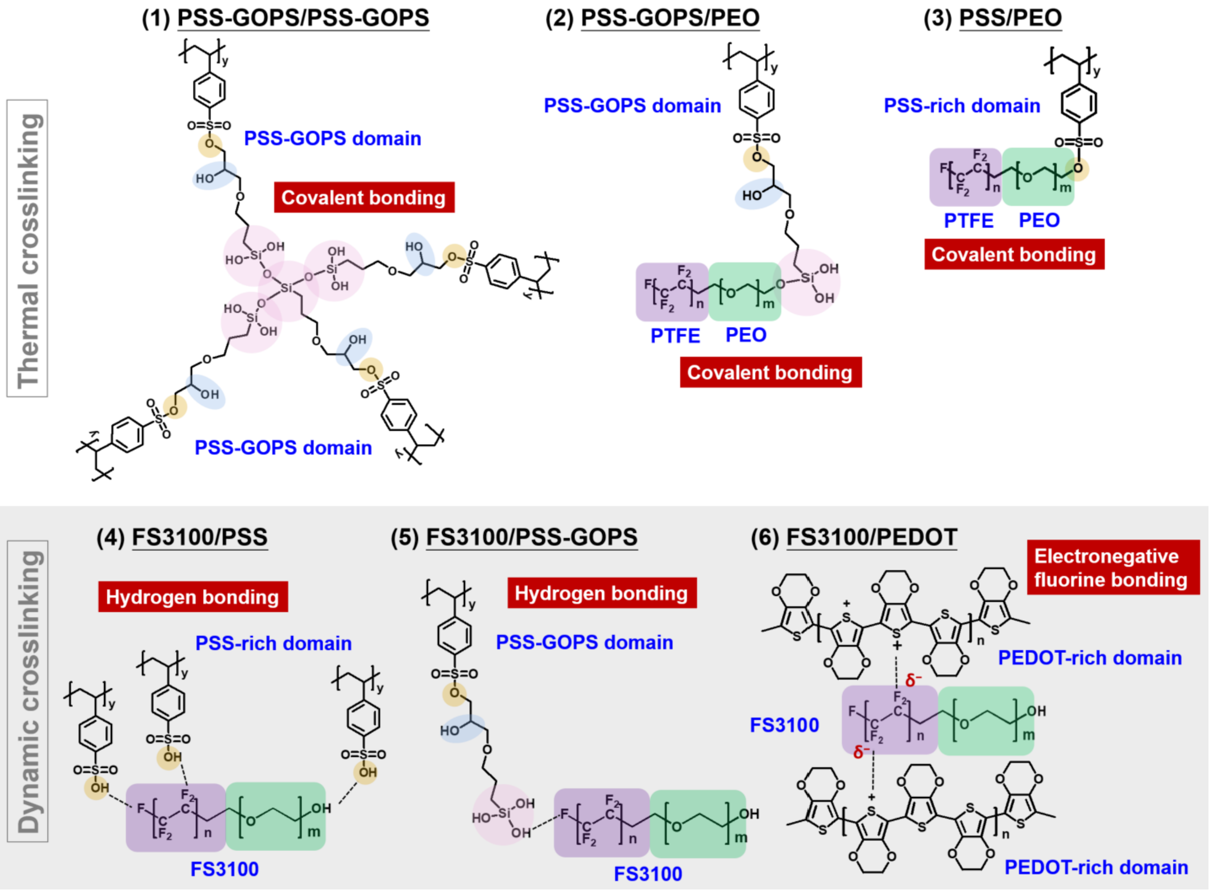

Figure S5. Potential crosslinking mechanisms of the **F10G0.5** composite film, including three thermal crosslinking pathways of (1) PSS-GOPS/PSS-GOPS, (2) PSS-GOPS/PEO, and (3) PSS/PEO interactions; as well as three dynamic crosslinking processes of (4) FS3100/PSS, (5) FS3100/PSS-GOPS, and (6) FS3100/PEDOT interactions.

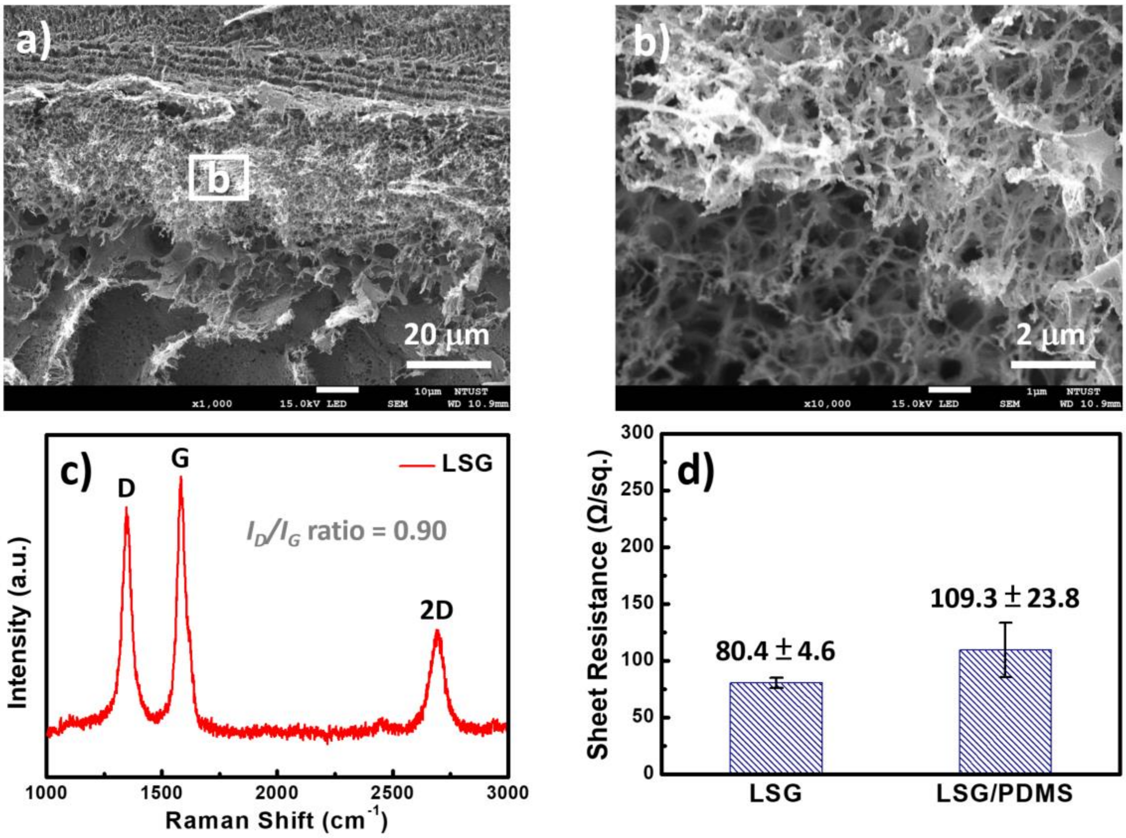

Figure S6. (a−b) SEM images of **LSG** derived from PI tape on glass substrate, with (b) providing an enlarged view of **LSG** from (a). (c) Raman spectrum of the **LSG** electrode. (d) Sheet resistances of **LSG** and **LSG/PDMS** electrodes.

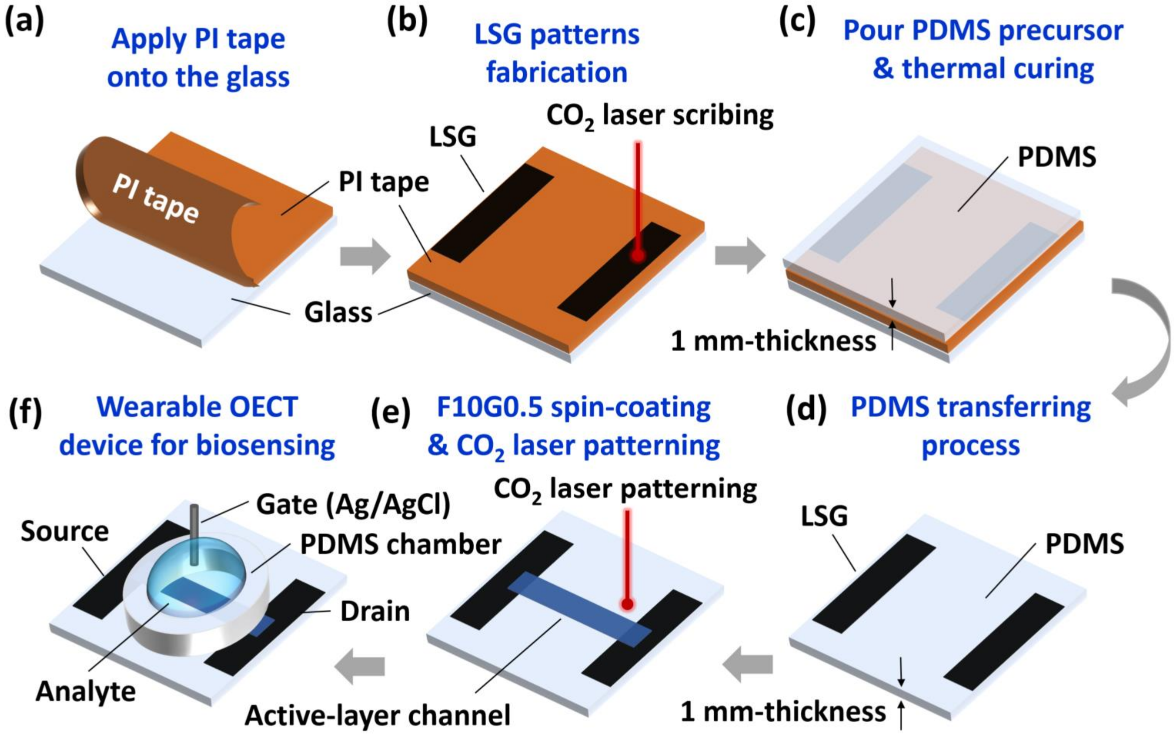

Figure S7. The fabrication process of wearable **F10G0.5**-based OECT device. (a) Apply PI

tape onto the glass substrate. (b) LSG patterns fabrication through the CO_2 laser scribing process. (c) Pour PDMS precursor and thermal curing process. (d) **LSG/PDMS** fabrication through the PDMS transferring process. (e) **F10G0.5/PDMS** fabrication through spin-coating and CO_2 laser patterning process. (f) Encapsulation of **F10G0.5**-based OECT device by a PDMS chamber.

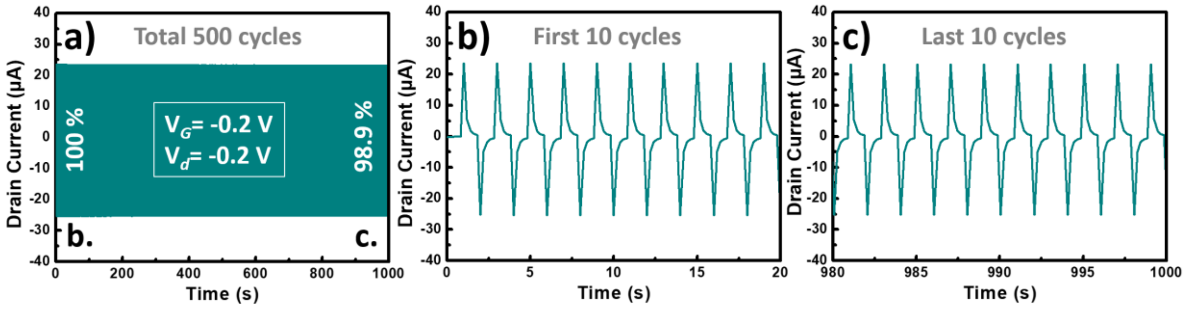

Figure S8. Long-term (I_D-*Time*) stability assessments conducted on F10G0.5-based OECTs in 1 × PBS (pH 7.4) buffer solution. The transient characteristics were measured in response to (a) 500 cycles of square V_G pulses (pulse voltage: −0.2 V; pulse width: 1 s; pulse period: 1 s), with V_D maintained at −0.2 V. This includes the (b) initial 10 cycles, and (c) last 10 cycles results.
